# The velvet worm brain unveils homologies and evolutionary novelties across panarthropods

**DOI:** 10.1186/s12915-021-01196-w

**Published:** 2022-01-25

**Authors:** Christine Martin, Henry Jahn, Mercedes Klein, Jörg U. Hammel, Paul A. Stevenson, Uwe Homberg, Georg Mayer

**Affiliations:** 1 Department of Zoology, Institute of Biology, University of Kassel, 34132 Kassel, Germany; 2 Institute of Materials Physics, Helmholtz-Zentrum hereon, 21502 Geesthacht, Germany; 3 Physiology of Animals and Behaviour, Institute of Biology, University of Leipzig, 04103 Leipzig, Germany; 4 Department of Biology, Animal Physiology, Philipps-Universität Marburg, 35043 Marburg, Germany; 5 Center for Mind, Brain and Behavior (CMBB), University of Marburg and Justus Liebig University Giessen, 35032 Marburg, Germany

**Keywords:** Nervous system, Central body, Mushroom body, Olfactory lobe, Neuroanatomy, Glossary

## Abstract

**Background:**

The evolution of the brain and its major neuropils in Panarthropoda (comprising Arthropoda, Tardigrada and Onychophora) remains enigmatic. As one of the closest relatives of arthropods, onychophorans are regarded as indispensable for a broad understanding of the evolution of panarthropod organ systems, including the brain, whose anatomical and functional organisation is often used to gain insights into evolutionary relations. However, while numerous recent studies have clarified the organisation of many arthropod nervous systems, a detailed investigation of the onychophoran brain with current state-of-the-art approaches is lacking, and further inconsistencies in nomenclature and interpretation hamper its understanding. To clarify the origins and homology of cerebral structures across panarthropods, we analysed the brain architecture in the onychophoran *Euperipatoides rowelli* by combining X-ray micro-computed tomography, histology, immunohistochemistry, confocal microscopy, and three-dimensional reconstruction.

**Results:**

Here, we use this detailed information to generate a consistent glossary for neuroanatomical studies of Onychophora. In addition, we report novel cerebral structures, provide novel details on previously known brain areas, and characterise further structures and neuropils in order to improve the reproducibility of neuroanatomical observations. Our findings support homology of mushroom bodies and central bodies in onychophorans and arthropods. Their antennal nerve cords and olfactory lobes most likely evolved independently. In contrast to previous reports, we found no evidence for second-order visual neuropils, or a frontal ganglion in the velvet worm brain.

**Conclusion:**

We imaged the velvet worm nervous system at an unprecedented level of detail and compiled a comprehensive glossary of known and previously uncharacterised neuroanatomical structures to provide an in-depth characterisation of the onychophoran brain architecture. We expect that our data will improve the reproducibility and comparability of future neuroanatomical studies.

**Supplementary Information:**

The online version contains supplementary material available at 10.1186/s12915-021-01196-w.

## Background

Onychophorans (velvet worms) constitute together with tardigrades (water bears) and arthropods (chelicerates, myriapods, crustaceans and hexapods) the Panarthropoda, with onychophorans being sister to either arthropods or arthropods plus tardigrades ([1–6]; see [7] for review of ecdysozoan relationships and additional phylogenetic hypotheses). Velvet worms are believed to have remained largely unchanged and inhabited terrestrial environments for over 300 million years [1, 8, 9]. Given their key phylogenetic position as one of the closest relatives to arthropods (sometimes referred to as Euarthropoda *sensu* Lankester [10]; discussed in Ortega-Hernández [11]), and their conserved morphology, onychophorans are widely regarded as indispensable for understanding the evolution of panarthropod organ systems (e.g. [12–19]).

Studies of the nervous system are often conducted to gain insights into evolutionary events [20–24]. For most major arthropod groups, in-depth investigations in recent times have led to a clear picture of the anatomical and functional organisation of their nervous systems. The nervous systems of onychophorans, in contrast, were studied mainly at the end of the nineteenth (e.g. [25, 26]) and beginning of the twentieth centuries (e.g. [27–31]), with only few recent exceptions (e.g. [13, 15, 32, 33]). Accordingly, the range of techniques used to investigate the onychophoran nervous system is largely limited to classical methods. Furthermore, most studies have focussed on specific aspects of the nervous system, such as neurogenesis and neural development [13, 15, 34–37] or on the localisation of specific structures and neurons using selective markers [14, 18, 33, 38–42]. Thus, a comprehensive overall picture is still lacking, especially in the light of the array of new methods currently available.

Unfortunately, further research on the onychophoran nervous system is currently hampered by inconsistencies in nomenclature and interpretations between different authors. A review of the literature reveals cases where a single structure is given different names by various authors. For example, the same part of the brain referred to as “arcuate body” by Strausfeld et al. [42] is named “central body” by Schürmann [32], “Zentralkörper” by Hanström [31], “corpus striatum” by Holmgren [27], and “bourrelet dorsal” by Saint-Remy [26]. In addition, the same term is sometimes applied to different structures, e.g. the term “pedunculus” to either a connection between mushroom body and central body [32], or a structure anterior to the mushroom body lobes [33], or even the mushroom body lobes themselves [43].

Similarly, there are numerous inconsistencies in descriptions of specific traits of the onychophoran brain. For example, the pathway of optic tracts and the number of optic neuropils as reported by Strausfeld et al. [33] and by Mayer [44]. In other cases, contradictions are evident even within a single study (e.g. “bridge” in [32]), or questionable characters are used to claim homology of structures with those in other animal groups (e.g. “frontal ganglion” in Cong et al. [45, 46], but see Mayer et al. [47] for an opposing view).

Finally, previous investigations of the onychophoran brain suggest that various structures remain to be described and characterised (e.g. an “anterior neuropil” referred to in [38, 48]), and emphasises that current understanding of the onychophoran brain is far from complete. Furthermore, the hitherto known architecture of the onychophoran brain has not been critically reappraised, although some features have already been questioned (e.g. the number of mushroom body lobes in [32]), while other features have been reported, but as yet not analysed in detail. For example, the onychophoran central body has been described to be stratified [26, 27, 42, 48], but the number and architecture of strata have not been addressed. Therefore, a revision of the onychophoran brain followed by a uniform designation of its components is needed in order to bring consensus to its anatomy and to clarify unresolved controversies.

In the current study, we report new insights into the structural organisation of the brain of the onychophoran *Euperipatoides rowelli* (Peripatopsidae), gained from using a range of specific markers against acetylated α-tubulin and the presynaptic protein synapsin in conjunction with high-resolution confocal microscopy. We then used the data from immunolabelled whole-mount preparations of the brain to three-dimensionally reconstruct its architecture and visualise previously unknown volumetric and spatial relationships among neurite regions. We supplement our data with three-dimensional (3D) reconstructions based on Azan-stained histological serial sections and X-ray micro-computed tomography (μCT). These analyses allowed us to gain an in-depth understanding of the onychophoran brain anatomy, revealing all major neuropil regions and allowing for their detailed characterisation. Together with neuroanatomical information available in the literature for the onychophoran nervous system, we compiled a glossary with proposed standardised terms and synonyms in order to bring consistency for future studies.

The multi-methodological approach enabled us to characterise in greater detail known neuropil regions and tracts and describe neuropil regions that were previously unknown. This in turn allows for a more comprehensive comparison of onychophoran brain structures to corresponding features in arthropods. Finally, we interpret our results to address several controversies surrounding the onychophoran brain. This reappraisal of the onychophoran nervous system should facilitate future comparative studies, and thereby shed light on the evolution of the nervous system in Panarthropoda and the brain structure in their last common ancestor.

## Results

**Fig. 1:**
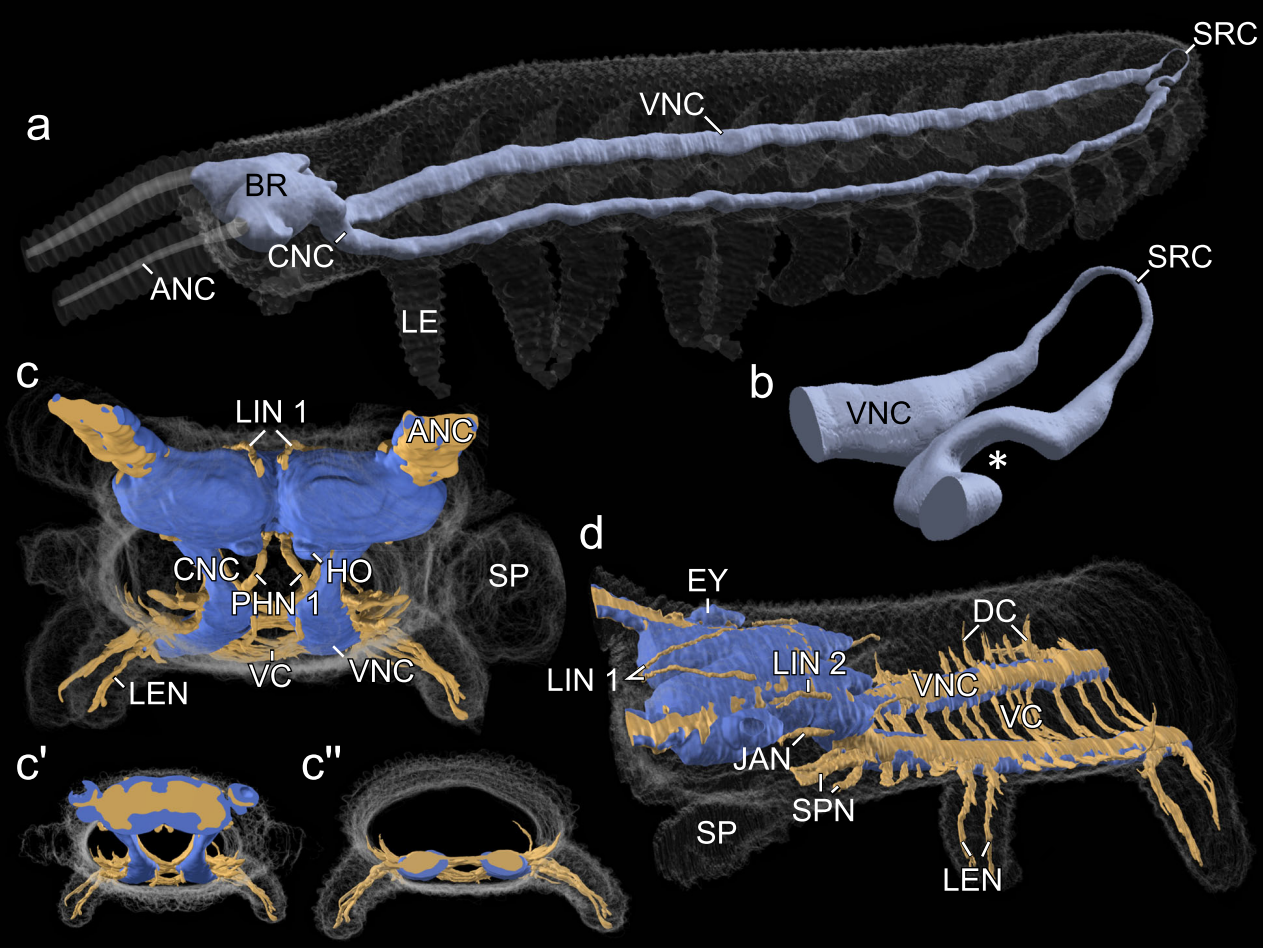
Organisation of the central nervous system of *E. rowelli*. 3D renderings based on SRμCT (**a, b**) and Azan staining (**c, d**). Dorsal is up in all images. **a** Overview. **b** Detail of suprarectal commissure. Asterisk indicates passage of genital duct under ventral nerve cord. **c, d** Frontal and lateral views of anterior part of nervous system. Cell body rind (blue) partly covers neuropils, fibre tracts, commissures and peripheral nerves (light-brown). **c', c”** Selected frontal slices from (**c**) at the level of the brain (**c'**) and ventral nerve cord (**c”**). ANC, antennal nerve cord; BR, brain; CNC, circumpharyngeal nerve cord; DC, dorsal commissures; EY, eye; HO, hypocerebral organ; JAN, jaw nerve; LEN, leg nerve; LE, leg; LIN 1, LIN 2, lip nerve1 and 2; PHN 1, pharyngeal nerve 1; SP, slime papilla; SPN, slime papilla nerves; SRC, suprarectal commissure; VC, ventral commissure; VNC, ventral nerve cord

**Fig. 2:**
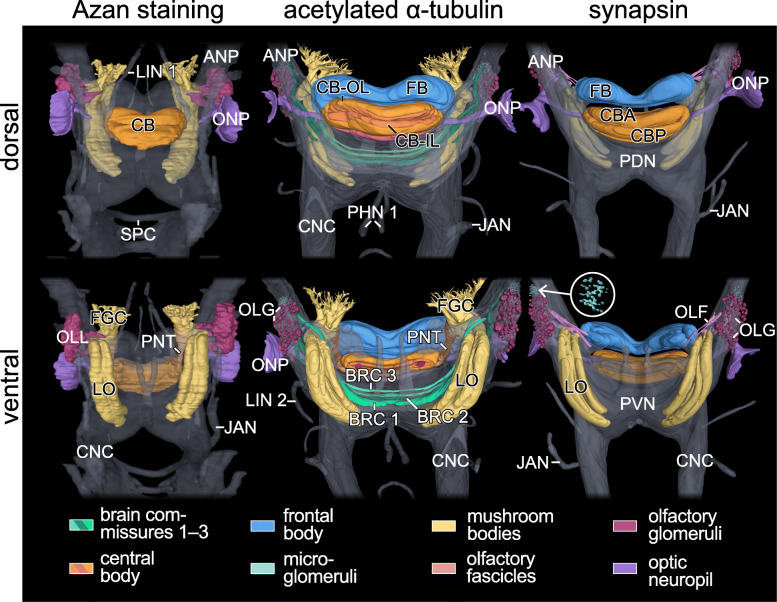
Architecture of cerebral structures in *E. rowelli* based on different labelling techniques. Three-dimensional reconstructions using a series of Azan-stained sections, anti-acetylated α-tubulin and anti-synapsin immunolabelling of entire brains in dorsal (top row) and ventral views (bottom row). Anterior is up in all images. Corresponding neuropil regions and fibre tracts are shown in same colours. Other protocerebral neuropil areas are displayed in grey. Inset (synapsin labelling in ventral view) shows magnified details of microglomeruli. Note that Azan staining was performed on the entire head, whereas anti-acetylated α-tubulin and anti-synapsin immunolabelling was carried out on dissected brains, resulting in slightly differing shapes of neuroanatomical structures. Not all structures were reconstructed using Azan-stained sections, such as the frontal body and small tracts and neuropils. ANP, antennal neuropil; BRC 1, BRC 2, BRC 3, brain commissure 1 to 3; CB, central body; CBA, anterior division of central body; CB-IL, inner lamina of central body; CB-OL, outer lamina of central body; CBP, posterior division of central body; CNC, circumpharyngeal nerve cord; FB, frontal body; JAN, jaw nerve; FGC, fascicles of globuli cells; LIN 1, LIN 2, lip nerve 1 and 2; LO, lobes of mushroom body; OLF, olfactory fascicle; OLG, olfactory glomeruli; OLL, olfactory lobe; ONP, optic neuropil; PDN, posterior dorsal neuropil; PHN 1, pharyngeal nerves 1; PVN, posterior ventral neuropil; PNT, ponticulus; SPC, subpharyngeal commissure

**Fig. 3:**
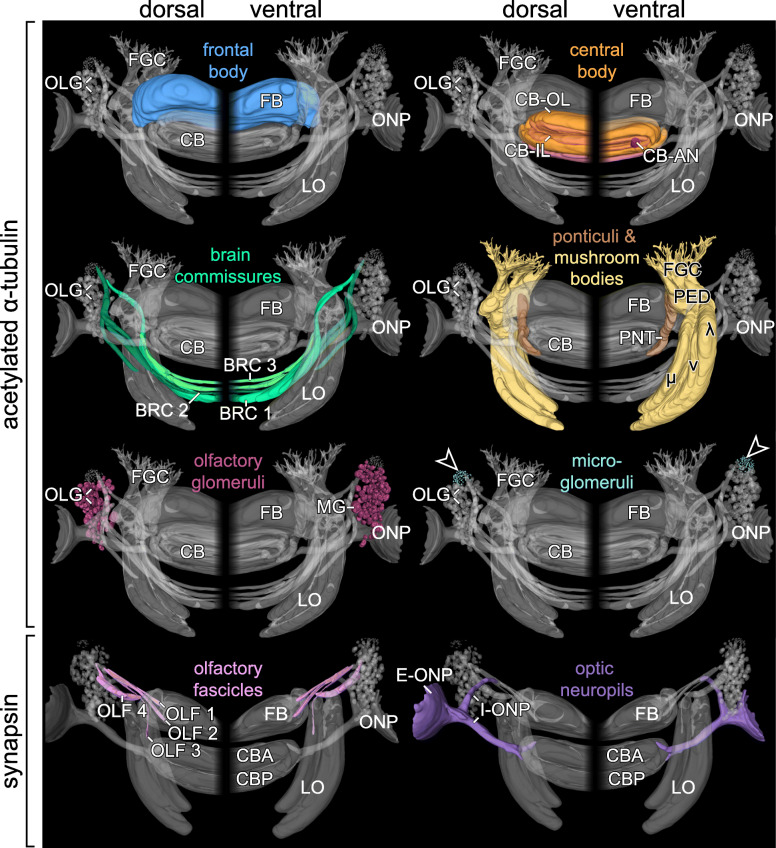
Comparison of individual commissures, tracts, lobes and neuropils in the brain of *E. rowelli*. Three-dimensional reconstructions in dorsal (left halves) and ventral views (right halves). Anterior is up in all images. Arrowheads indicate position of microglomeruli. BRC 1, BRC 2, BRC 3, brain commissure 1 to 3; FGC, fascicles of globuli cells; CB, central body; CBA, anterior division of central body; CB-AN, auxiliary neuropil of central body; CB-IL, inner lamina of central body; CB-OL, outer lamina of central body; CBP, posterior division of central body; CNC, circumpharyngeal nerve cord; E-ONP, extracerebral part of optic neuropil; FB, frontal body; I-ONP, intracerebral part of optic neuropil; λ, lateral or λ-lobe; LO, lobes of mushroom body; MG, macroglomerulus; μ, median or μ-lobe; ν, ventral or ν-lobe; OLF 1, OLF 2, OLF 3, OLF 4, olfactory fascicles 1 to 4; OLG, olfactory glomeruli; ONP, optic neuropil; PED, pedunculus; PNT, ponticulus

**Fig. 4:**
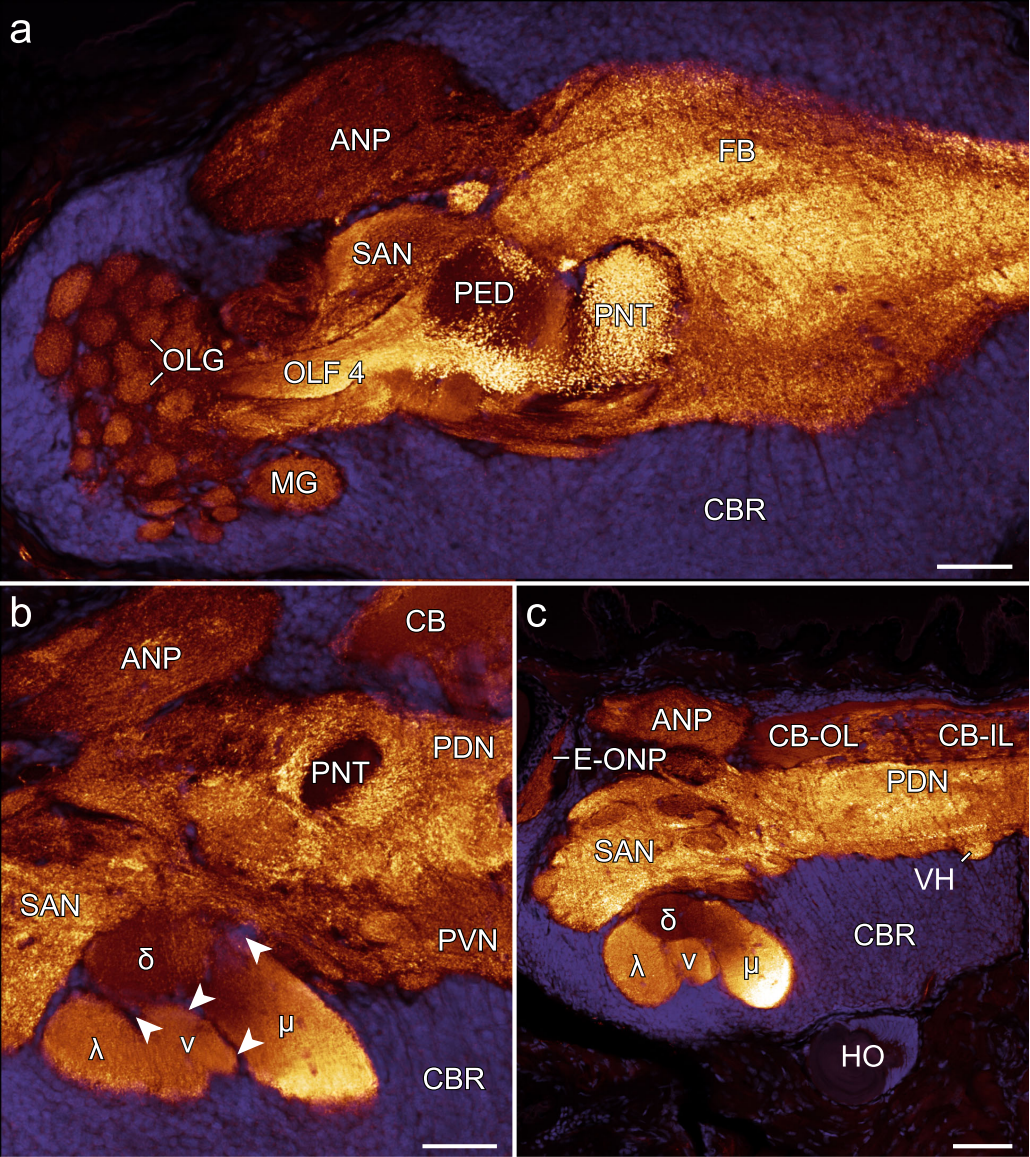
Organisation of brain neuropils in *E. rowelli* revealed by anti-synapsin immunolabelling. Confocal laser scanning micrographs of frontal vibratome sections. Dorsal is up in all images. Anti-synapsin immunoreactivity (glow) and DNA labelling (blue) in anterior (**a**), median (**b**), and posterior sections (**c**) through the left brain hemisphere. Note strong immunoreactivity in the ponticulus in **a** and near lack thereof in **b**. Also note strongest immunoreactivity in μ-lobe as opposed to weakest immunoreactivity in δ-lobe (**c**) of mushroom body. Arrowheads (**b**) point to discontinuous row of somata between mushroom body lobes. ANP, antennal neuropil; CB, central body; CB-IL, inner lamina of central body; CB-OL, outer lamina of central body; CBR, cell body rind; δ, dorsal or δ-lobe of mushroom body; E-ONP, extracerebral part of optic neuropil; FB, frontal body; HO, hypocerebral organ; λ, lateral or λ-lobe of mushroom body; μ, median lobe or μ-lobe of mushroom body; MG, macroglomerulus; OLF 4, olfactory fascicle 4; OLG, olfactory glomeruli; PDN, posterior dorsal neuropil; PNT, ponticulus; SAN, subantennal neuropil; v, ventral or v-lobe of mushroom body; VH, ventral horn; PVN, posterior ventral neuropil. Scale bars: 50 μm

**Fig. 5:**
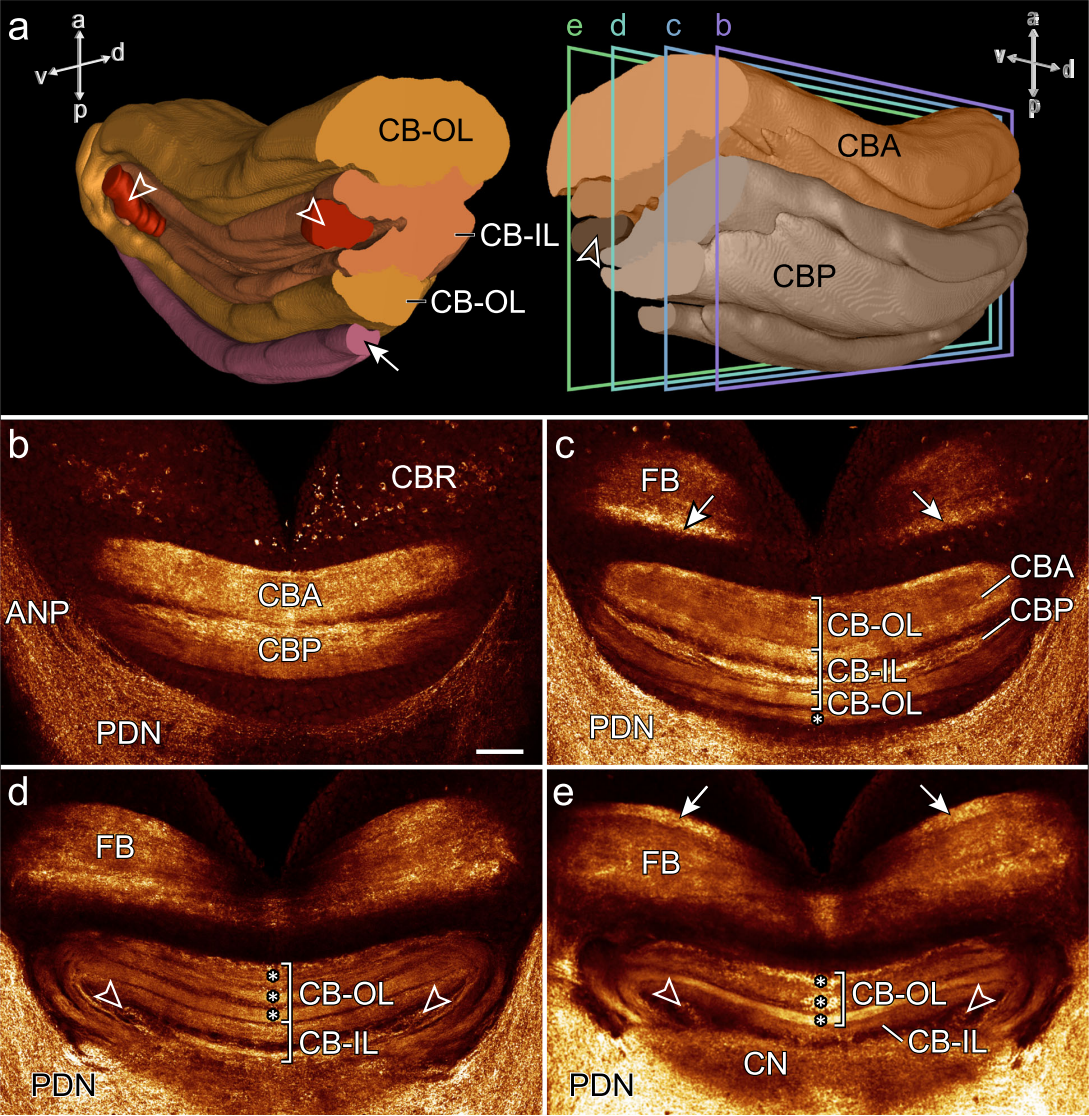
Organisation of the central body and frontal body in *E. rowelli*. Three-dimensional reconstructions based on anti-acetylated α-tubulin immunolabelling (**a**) and horizontal optical sections of confocal micrographs of a brain stained with an antibody against synapsin (**b**–**e**). Anterior is up in all images. Arrow (**a**) indicates additional posterior stratum of posterior division and arrowheads point to auxiliary neuropils. Rectangles in (**a**) indicate planes of sections in (**b**–**e**). **b, c** Dorsal part of central body showing anterior and posterior divisions. Asterisk in **c** indicates additional stratum of posterior division. **d, e** Ventral part of central body illustrating inner lamina enclosing paired auxiliary neuropils (arrowheads) and outer lamina with additional strata on its ventral side (asterisks). Note frontal body with a posterior, dorsal stratum (arrows in **c**) and an anterior, ventral stratum (arrows in **e**). a, anterior; ANP, antennal neuropil; CBA, anterior division of central body; CB-IL, inner lamina of central body; CB-OL, outer lamina of central body; CBP, posterior division of central body; CBR, cell body rind; CN, central neuropil; d, dorsal; FB, frontal body; p, posterior; PDN, posterior dorsal neuropil; v, ventral. Scale bar: 50 μm

**Fig. 6:**
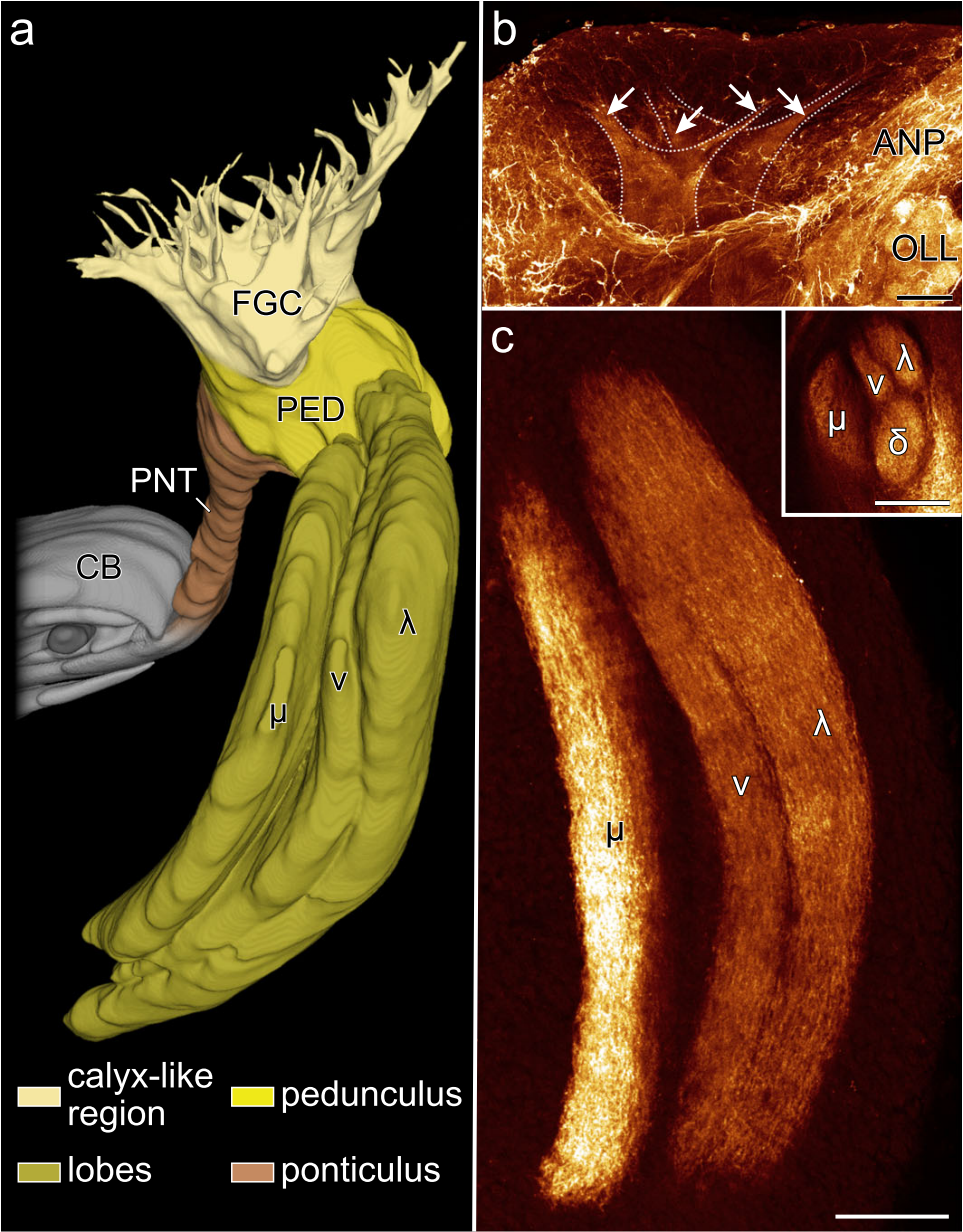
Organisation of mushroom body in *E. rowelli*. Three-dimensional reconstruction based on anti-acetylated α-tubulin immunolabelling (**a**) and horizontal optical sections from stacks of confocal micrographs of brains stained against acetylated α-tubulin (**b**) and synapsin (**c**). Anterior is up and median is left in all images. **a** Volume rendering of mushroom body in ventral view. Note connection with central body via ponticulus. **b** Detail of fascicles of globuli cells with two main branches (dotted line) giving rise to four digitiform branches (arrows). **c** Detail of the four lobes, all of which are sectioned in inset. Note strongest synapsin immunoreactivity along μ-lobe. ANP, antennal neuropil; CB, central body; δ, dorsal or δ-lobe; FGC, fascicles of globuli cells; λ, lateral or λ-lobe; μ, median or μ-lobe; OLL, olfactory lobe; PED, pedunculus; PNT, ponticulus; v, ventral or v-lobe. Scale bars: 50 μm

**Fig. 7:**
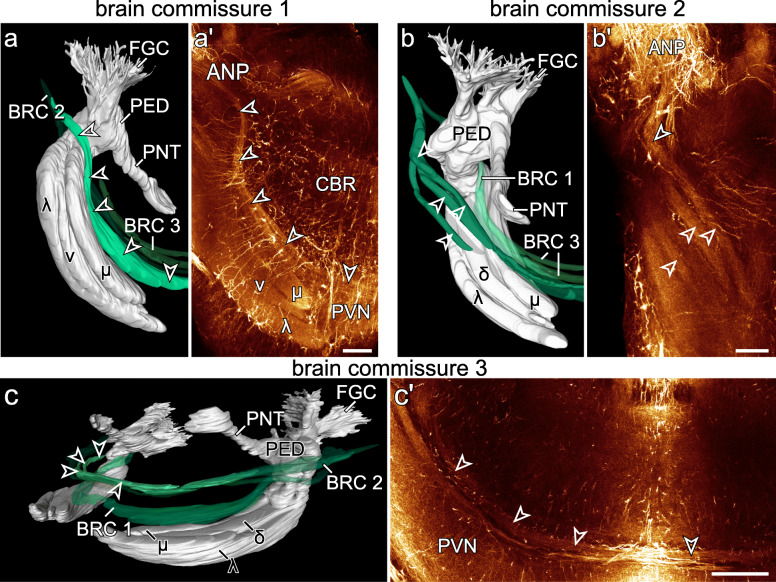
Organisation of brain commissures in *E. rowelli*. Three-dimensional reconstructions based on anti-acetylated α-tubulin immunolabelling (**a**–**c**) and horizontal optical sections of confocal micrographs of brains stained for acetylated α-tubulin (**a'**–**c'**). Anterior is up in (**a**, **a'**, **b**, **b'** and **c'**), top right corner in **c** and dorsal is up in **c**. Reconstructed brain commissures are depicted in green and shown in spatial relation to mushroom body (grey in **a**, **b** and **c**). Brain commissures 1 (**a**; ventral view) and 2 (**b**; dorsal view) originate from antennal neuropil and proceed adjacent to mushroom body lobes, whereas brain commissure 3 (**c**; posterolateral view) proceeds dorsal to mushroom body lobes. Arrowheads point to corresponding commissures. ANP, antennal neuropil; BRC 1, BRC 2, BRC 3, brain commissure 1 to 3; CBR, cell body rind; δ, dorsal or δ-lobe of mushroom body; FGC, fascicles of globuli cells; λ, lateral or λ-lobe of mushroom body; μ, median or μ-lobe of mushroom body; PED, pedunculus; PNT, ponticulus; PVN, posterior ventral neuropil; v, ventral or v-lobe of mushroom body. Scale bars: 50 μm

**Fig. 8:**
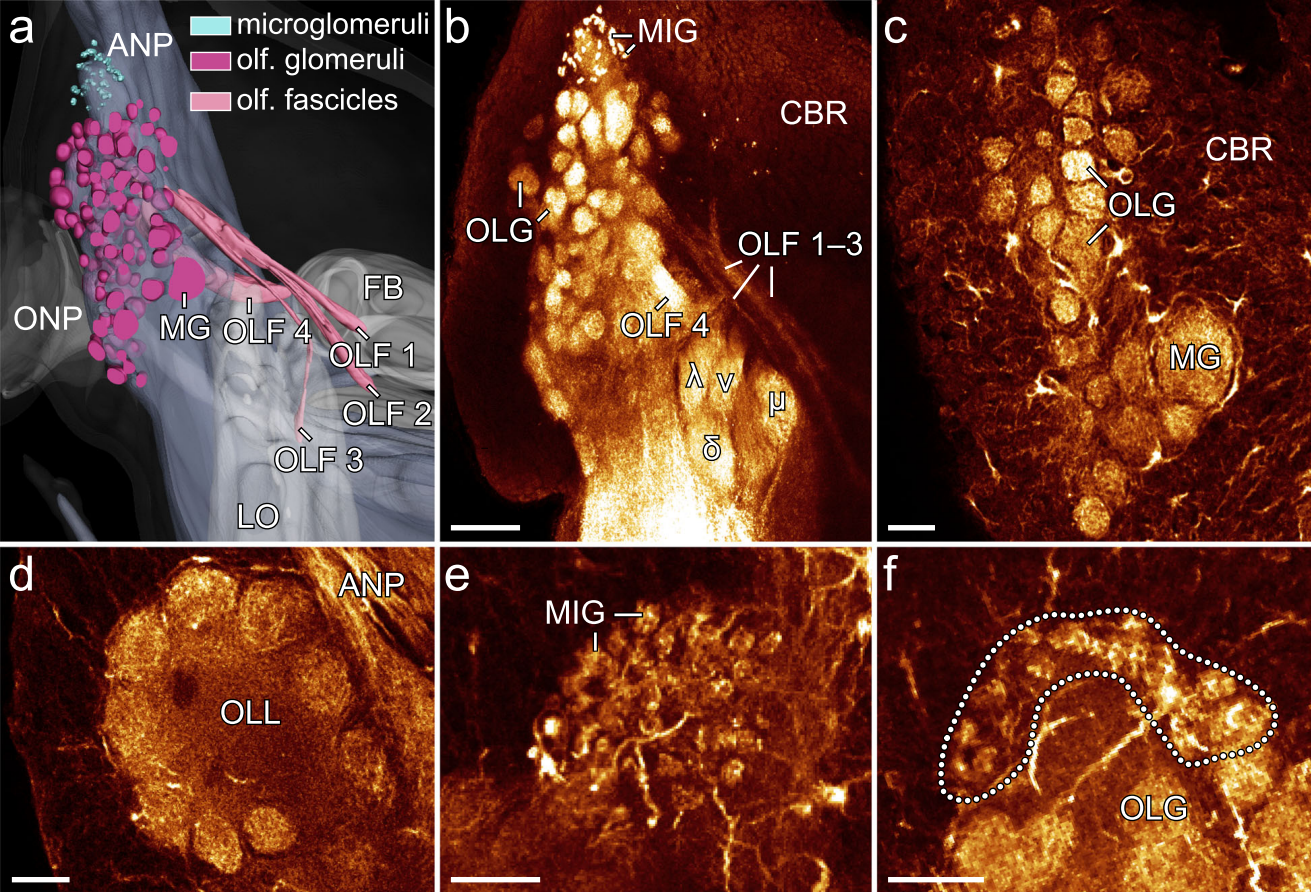
Organisation of olfactory and glomerular structures in *E. rowelli*. Three-dimensional reconstruction based on anti-synapsin immunolabelling (**a**) and horizontal optical sections of confocal micrographs of brains stained for synapsin (**b**) and acetylated α-tubulin (**c**–**f**). Anterior is up in all images. **a, b** Olfactory fascicles 1–3 adjacent to mushroom body’s lobes. Olfactory fascicle 4 connects to v- and δ-lobe (see Additional file 10: Data S5 and Additional file 11: Data S6). **a**–**d** Size and arrangement of olfactory glomeruli (**a** and **c**). Note their similar size and presence of a macroglomerulus. **e, f** Cap-like arrangement of microglomeruli anterior to olfactory glomeruli. ANP, antennal neuropil; CBR, cell body rind; δ, dorsal or δ-lobe of mushroom body; FB, frontal body; λ, lateral or λ-lobe of mushroom body; LO, lobes of mushroom body; MG, macroglomerulus; MIG, microglomeruli; μ, median or μ-lobe of mushroom body; OLF 1, OLF 2, OLF 3, OLF 4, olfactory fascicle 1 to 4; OLG, olfactory glomerulus; OLL, olfactory lobe; ONP, optic neuropil; v, ventral or v-lobe of mushroom body. Scale bars: 50 μm (**b** and **c**) and 20 μm (**d**–**f**)

**Fig. 9:**
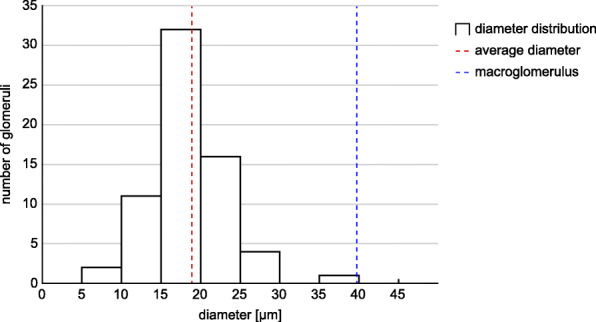
Diameter range of glomeruli in *E. rowelli*. The diameters of 65 out of a total of ~ 80 glomeruli of one brain hemisphere were measured. Dashed red line indicates average diameter of glomeruli; dashed blue line demarcates diameter of macroglomerulus

**Fig. 10:**
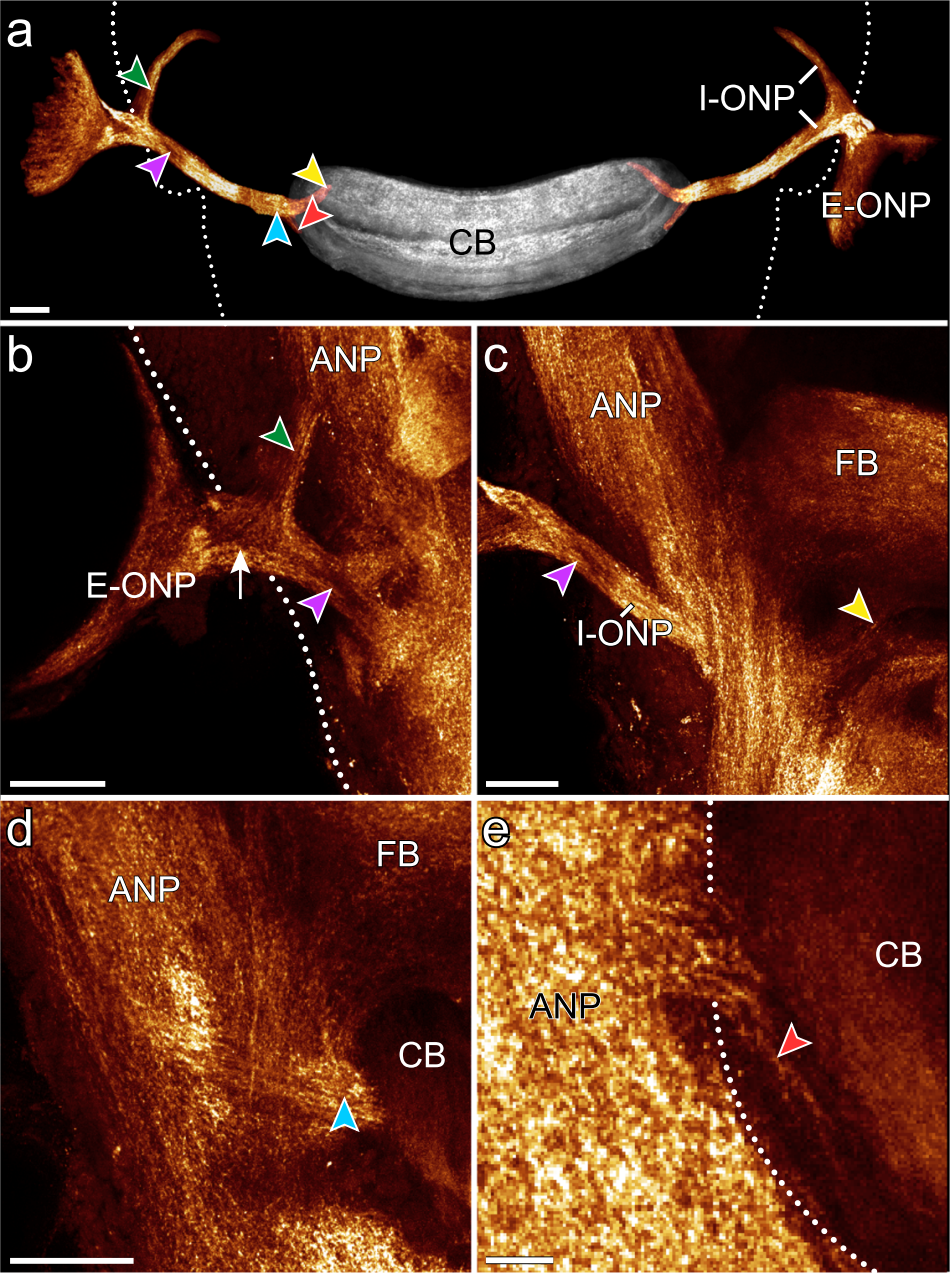
Visual pathways in *E. rowelli*. Horizontal optical sections of confocal micrographs of brains immunolabelled for synapsin. Anterior is up in all images. **a** Spatial relationship between optic neuropils (glow) and central body (grey). These structures were manually segmented in Amira and extracted from original image stack using Fiji (“Image calculator” with “AND” as operational mode). Unmodified dataset is provided in Additional file 11: Data S6. Dotted line (**a** and **b**) indicates outline of brain. Coloured arrowheads point to corresponding positions in (**b**–**d**). **b**–**d** Details of optic neuropil in ventral (**b**) to dorsal (**d**) series of sections. Bottleneck-shaped extracerebral part of optic neuropil connects to brain (arrow in **b**). Note bifurcations of intracerebral part of optic neuropil in its distal and proximal regions. Note also input into additional posterior stratum of posterior division of central body (**e**). Dotted line indicates lateral border of central body in **e**. ANP, antennal neuropil; CB, central body; E-ONP, extracerebral part of optic neuropil; FB, frontal body; I-ONP, intracerebral part of optic neuropil. Scale bars: 50 μm (**a–d**) and 10 μm (**e**)

**Fig. 11:**
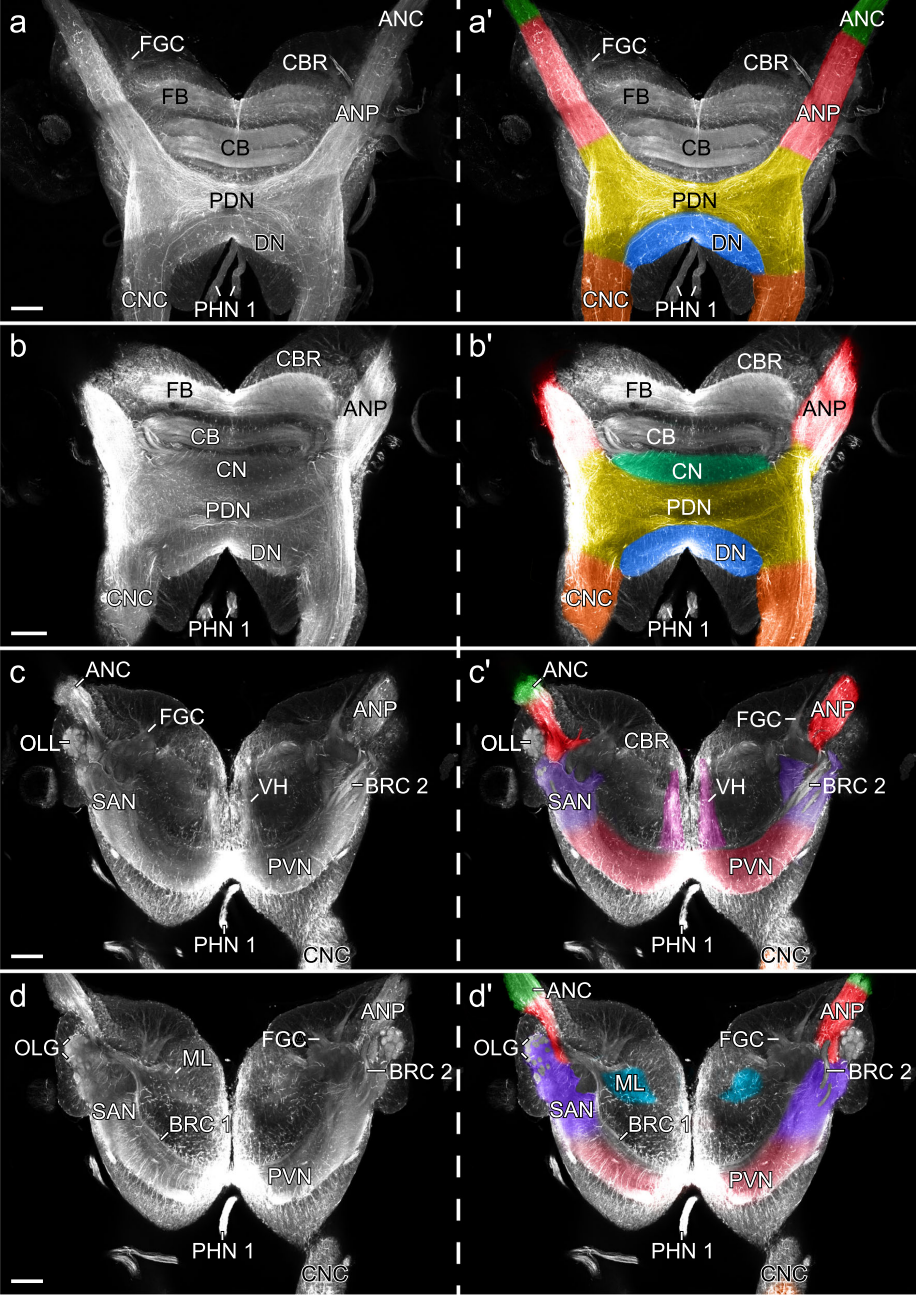
Organisation and characterisation of brain neuropils in *E. rowelli.* Horizontal optical sections of confocal micrographs of brains immunolabelled for acetylated α-tubulin. Anterior is up in all images. Coloured brain areas of largely fused neuropils and lobes (right) from dorsal (**a**) to ventral (**d**) series of sections. ANC, antennal nerve cord; ANP, antennal neuropil; BRC 1 and 2, brain commissure 1 and 2; CB, central body; CBR, cell body rind; CN, central neuropil; CNC, circumpharyngeal nerve cord; DN, deutocerebral neuropil; FB, frontal body; FGC, fascicles of globuli cells; ML, median lobe; OLG, olfactory glomeruli; OLL, olfactory lobe; PHN 1, pharyngeal nerves 1; PDN, posterior dorsal neuropil; PVN, posterior ventral neuropil; SAN, subantennal neuropil; VH, ventral horn. Scale bars: 100 μm

**Fig. 12:**
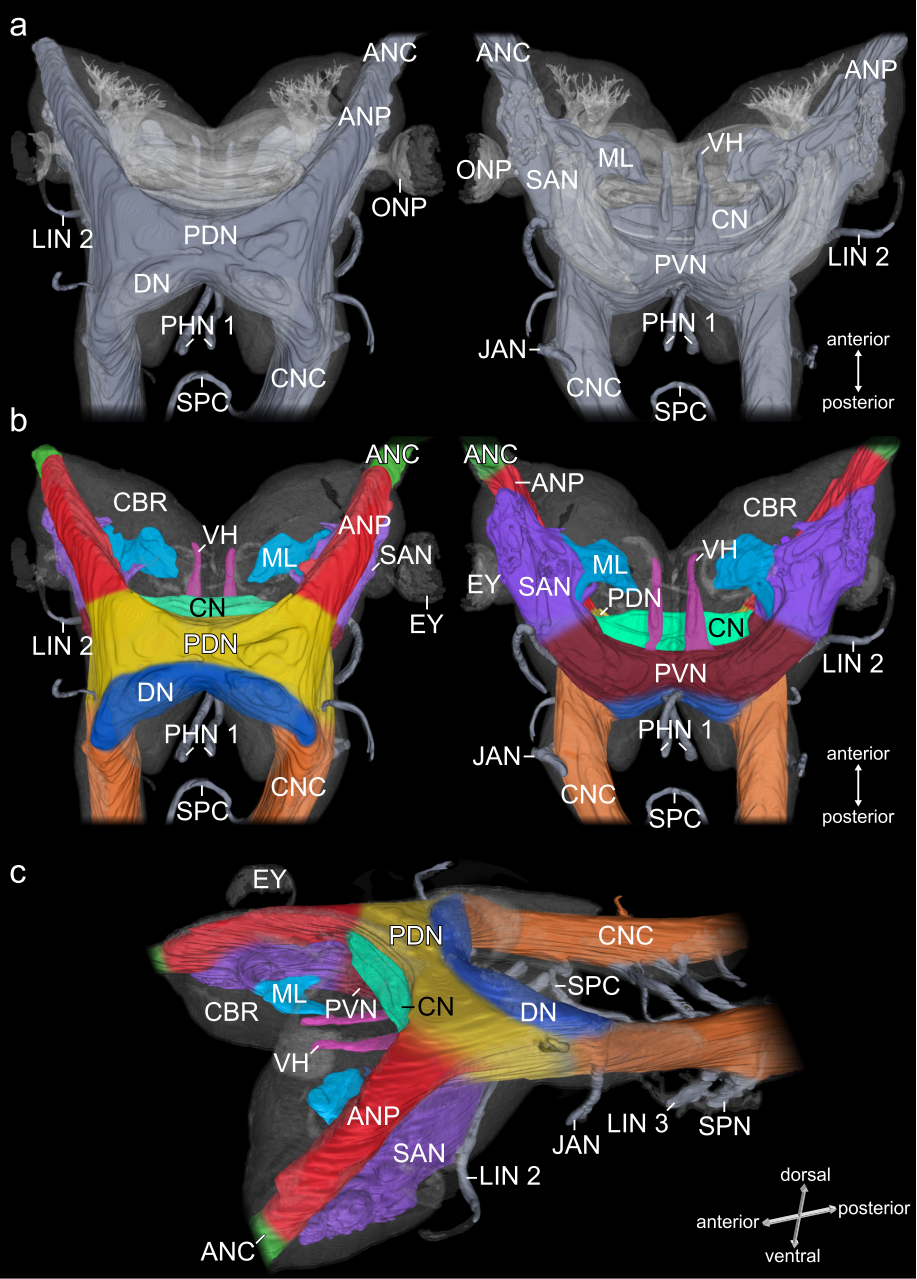
Organisation and characterisation of brain neuropils in *E. rowelli*. Three-dimensional reconstruction based on anti-acetylated α-tubulin immunolabelling. **a** Brain in dorsal (left) and ventral views (right) illustrating largely fused neuropils and lobes (bluish grey). For clarity the mushroom bodies, olfactory lobes, frontal neuropil, visual pathways and central body are collectively shown in semi-transparent light grey. **b** Colour-coded brain areas in dorsal (left) and ventral views (right). **c** Same as **b** in anterolateral view. ANC, antennal nerve cord; ANP, antennal neuropil; CBR, cell body rind; CN, central neuropil; CNC, circumpharyngeal nerve cord; DN, deutocerebral neuropil; EY, eye; JAN, jaw nerve; LIN 2, LIN 3, lip nerves 2 and 3; ML, median lobe; ONP, optic neuropil; PHN 1, pharyngeal nerves 1; PDN, posterior dorsal neuropil; PVN, posterior ventral neuropil; SAN, subantennal neuropil; SPC, subpharyngeal commissure; SPN, slime papilla nerve; VH, ventral horn

We used a multiscale approach to gain a broader and detailed picture of the brain structure in the onychophoran *E. rowelli* (Figs. 1, 2, 3, 4, 5, 6, 7, 8, 9, 10, 11, 12 and Additional file 1: Fig. S1, Additional file 2: Fig. S2, Additional file 3: Fig. S3, Additional file 4: Fig. S4, Additional file 5: Fig. S5, Additional file 6: data S1, Additional file 7: Data S2, Additional file 8: Data S3, Additional file 9: Data S4, Additional file 10: Data S5, Additional file 11: Data S6)*.* X-ray μCT scans of specimens contrasted with osmium tetroxide and ruthenium red proved useful for visualising the overall shape of the onychophoran central nervous system within the whole animal. In addition, histological sections of entire heads in conjunction with Azan staining provided detailed structural information on the central nervous system with its individual neuropil areas without dissection artefacts. Finally, an in-depth picture of brain architecture was provided by synapsin immunolabelling of dissected brains to reveal synaptic terminals and, hence, details of neuropil areas, while acetylated α-tubulin immunolabelling showed pathways traversed by fibres of the nervous system.

Neuroanatomical terms suggested herein as a standard nomenclature for studies of the onychophoran nervous system are preceded by an arrow (→). The corresponding explanations, definitions and synonyms are compiled in a glossary (Table 1). Whenever possible, the nomenclature follows the terms and definitions suggested by Ito et al. [49] for insects and Richter et al. [20] for invertebrates in general. Note that corresponding terms do not necessarily imply homology with homonymous (i.e. like-named) structures in arthropods and other animals.

**Table 1:** Glossary of neuroanatomical terms for studies of Onychophora. Terms that are highlighted in bold and preceded by arrows are defined in corresponding fields. Novel introduced terms are indicated by asterisks (*). Languages of foreign synonyms are specified in square brackets. Note that correspondent terms do not necessarily imply homology with homonymous structures in arthropods and other animals. Whenever possible, the nomenclature follows the terms and definitions suggested by Ito et al. [49] for insects and Richter et al. [20] for invertebrates in general

Suggested term for neuroanatomical structure (of one brain hemisphere or body side)	Reference(s) for suggested neuroanatomical term	Abbreviation	Figures	Description	Synonyms (plural and singular forms were adapted to terminology used herein)	Reference(s) for synonyms
Antennal nerve cord	[33]	ANC	1a, c, 11a, c, 12a–c, 13 and Additional file 1: Fig. S1a–c and Additional file 5: Fig. S5a–d	Prominent neuropil and bundle of fibres supplying each antenna; it begins in antennal tip, traverses antennal shaft and continues as **→antennal neuropil** in **→brain**; it is true neuropil, as it contains synapses revealed by transmission electron microscopy [32] and anti-synapsin immunolabelling (this study), and shows medullary organisation, as it is accompanied by somata [13]; this combination of neuropil and medullary organisation speaks against designation of this structure as nerve but rather as nerve cord	Antennalnerv [German]	[27, 28, 31, 50–53]
					Antennal nerve	[15, 32, 34, 40, 43, 48, 54–68]
					Antennal neuropil	[57]
					Antennal tract	[18, 32, 38, 39, 43, 47, 48, 69–72]
					Antennalstrang [German]	[27, 28, 30, 31, 51]
					Antennary nerve	[25, 73–76]
					Antennennerv [German]	[73, 74]
					Anterior appendage tract	[13]
					Dorsolateral lobe/horn	[25]
					Frontal appendage nerve	[45]
					Frontal appendage nerve bundle	[75]
					Fühlernerv [German]	[76]
					lobe antennaire [French]	[26]
					Lobus antennalis + Tractus antennalis [Latin]	[27]]
					nerf antennaire [French]	[26, 77]
					Nerve strand of the antenna	[65]
					nervo antenna [Portuguese]	[78]
					Nervus antennalis [Latin]	[30, 53, 79]
					Tentakelnerv [German]	[80]
Antennal neuropil	[57]	ANP	2, 4a–c, 5b, 6b, 7a', b', 8a, d, 10b–e, 1a–d, 12a–c, 13 and Additional file 1: S1a–c, Additional file 2: Fig. S2b and Additional file 5: Fig. S5a, b, d	Dorsolateral neuropil in **→brain**, part of each brain hemisphere; it is continuation of **→antennal nerve cord** within brain; it is fused ventrally with **→subantennal neuropil** and posteriorly with **→posterior dorsal neuropil** and sends off fibres to **→auxiliary neuropil** of **→central body**	Antennalfaserstrang [German]	[51]
					Antennal lobe	[32, 34, 39, 43, 47, 48, 54, 56, 65, 69]
					Antennal nerve	[15, 34, 40, 48, 55, 57–59, 66–68]
					Antennal nerve cord	[33]
					Antennalstrang [German]	[27, 28, 30, 31, 51]
					Antennal tract	[18, 34, 38, 39, 43, 47, 56, 59, 69–72]
					Antennennerv [German]	[52]
					Anterior appendage tract	[13]
					crête dorso-laterale [French]	[26]
					Dorsolateral lobe/horn	[25, 81]
					Frontal appendage ganglion	[45, 75]
					Lateral brain neuropil	[17, 48, 69]
					lobe antennaire [French]	[26]
					Lobus antennalis + Tractus antennalis [Latin]	[27]
					Nerve cord of pre-ocular frontal appendage	[45]
					Postglomerular lateral neuropil	[13]
					Ventrolateral horn	[25]
Anterior division (of central body)	[38]	CBA	2, 3, 5a, b and 13	Anterior subunit of **→central body**, which contains looping neurites forming **→outer lamina** and **→inner lamina**; fuses laterally with **→posterior division** at ventral part of central body	Anterior lamina	[17, 48, 69]
					Lame antérieure du bourrelet dorsal [French]	[26]
					lamina anterior [Latin]	[32, 54, 65]
					Lamina anterior des Zentralkörpers [Latin + German]	[31]
					vordere Lamelle des gestreiften Körpers [German]	[28]
					vorderer Zentralkörperteil [German]	[27]
Auxiliary neuropil*	–	CB-AN	3, 13 and arrowheads in 5a, d, e	Small cylindrical neuropil located on ventral side of **→central body** and enclosed by **→inner lamina**; it receives fibres from **→antennal neuropil**	–	–
Brain	[13, 17, 25, 32–34, 37–40, 42, 43, 45, 47, 48, 54–57, 59, 61–63, 65–72, 75, 81–98]	BR	1a and Additional files 1: Fig. S1a–c	Anterodorsal part of central nervous system consisting of left and right brain hemispheres, including cell body rind and brain neuropils; it is composite, bipartite structure (=syncerebrum *sensu* [20]) composed of **→protocerebrum** (belonging to 1st body segment) and **→deutocerebrum** (belonging to 2nd body segment); it accordingly innervates two pairs of cephalic appendages, including antennae (via **→ antennal nerve cords**), and jaws (via **→ jaw nerves**); it is associated posteriorly with **→circumpharyngeal nerve cords**	Bilobed cephalic ganglion	[99]
					Bilobed supra-oesophageal ganglia	[63]
					Cephalic ganglia	[100]
					Cephalic ganglia [Russian: головные узлы]	[101]
					Cerebral ganglion	[102]
					cerebro [Portuguese]	[78]
					cerveau [French]	[26, 103, 104]
					ganglion céphalique [French]	[101, 102]
					ganglions céphaliques [French]	[26]
					ganglions sus-œsophagiennes [French]	[105]
					ganglions cérébroïdes [French]	[106]
					gánglios cerebroídes [Spanish]	[107]
					Gehirn [German]	[27, 28, 30, 31, 51–53, 73, 79, 108–111]
					Kopfganglion [German]	[112]
					Oberschlundganglion [German]	[51]
					Oberschlundganglionmasse [German]	[29]
					paarige Gehirnganglien [German]	[76]
					paarige Oberschlundganglien [German]	[50]
					paarige Schlundganglien [German]	[76]
					paire de gros ganglions sus-œsophagiens [French]	[77]
					paired cerebral masses	[100]
					Schlundganglion [German]	[113]
					supra-oesophageal ganglia	[82, 114]
					supra-œsophageal ganglia	[25, 81, 115]
					supra-oesophageal ganglion	[63]
Brain commissures 1–3*	–	BRC 1–3	2, 3, 7a–c, 11c, d, 13 and arrowheads and arrows in Additional file 3: Fig. S3a–f	Bundles of contralateral fibres crossing **→protocerebrum**; brain commissures 1 and 2 link both **→antennal neuropils**; they, circumvent **→pedunculus** and follow **→lobes** of **→mushroom body** to **→brain** midline where they cross to contralateral brain hemisphere; brain commissure 3 is situated anterior to brain commissures 1 and 2 and links both **→subantennal neuropils**; it then splits in two branches before proceeding to contralateral brain hemisphere	Accessory lobes	[32, 33, 48, 69]
					Accessory stalks	[32, 54, 65]
					Heterolateral commissures	[33, 42]
					Heterolateral mushroom body lobes	[33]
					Nebentrabekel [German]	[30]
Central body	[17, 32, 38–40, 48, 54, 56, 62, 65, 67–69, 84, 86]	CB	2, 3, 4b, 5a–e, 6a, 10a, d, e, 11a, b and 13	Dorsalmost, midline neuropil located posterior to **→frontal body**; it is arcuate in shape and consists of **→outer lamina** and **→inner lamina**, resulting in stratified appearance; inner lamina encloses**→auxiliary neuropils**; **→anterior division** and **→posterior division** fuse at ventral part of central body; posterior division is associated posteriorly with additional posterior stratum, which receives fibres from **→optic neuropil**	Arcuate body	[33, 42, 57, 84, 96]
					bourrelet dorsal [French]	[26]
					Central complex	[75]
					corpo central [Portuguese]	[78]
					corpo estriado [Portuguese]	[78]
					corpus centrale [Latin]	[43]
					corpus striatum [Latin]	[32, 54, 56]
					Corpus striatum [Latin]	[27, 28, 30]
					dorsal cap	[90]
					gestreifter Körper [German]	[27, 28, 30]
					Posterodorsal lobe	[25]
					Zentralkörper [German]	[27, 30, 31, 52, 53, 110, 111]
Central neuropil	[13, 17, 32, 34, 37, 38, 47, 48, 55, 56, 59, 65, 69, 70, 86, 98]	CN	5e, 11b, 12a–c and Additional file 5: Fig. S5a	Midline-spanning neurite region, which is located anterior to **→posterior dorsal neuropil** and **→posterior ventral neuropil** and receives fibres from **→antennal neuropil**	Central brain neuropil	[15, 48, 55, 59, 70]
					Central fibrous mass	[56]
					Central lobe	[25, 81, 82]
					Median lobe	[25]
					Zentralfasermasse [German]	[51]
Circumpharyngeal nerve cord*	–	CNC	1a, 2, 11a–d, 12a–c, 13 and Additional file 1: Fig. S1a, c and Additional file 5: Fig. S5a–d	Dorsoventral medullary nerve cord connecting brain hemispheres with **→ventral nerve cords**; it entirely lacks **→dorsal commissures** and is medially associated only with **→subpharyngeal commissure**; it laterally gives rise to **→slime papilla nerves** that lie posterior to **→lip nerve 3**, and anterior to **→salivary gland nerve**; circumpharyngeal nerve cord consists of outer cell body rind and inner neuropil	Circumoesophageal connective	[13, 32, 34, 56, 66, 91, 97]
					Circumpharyngeal connective	[92]
					Circumpharyngeal medullary connective	[68]
					commissure œsophagienne [French]	[26]
					Connecting cord	[38, 39, 55, 69, 86]
					Connecting piece	[40, 48, 72]
					Connective of the brain	[93]
					Connective to the ventral nerve cord	[65]
					Hinterschlundkommissur [German]	[31]
					Konnektivstrang der Schlundganglien [German]	[27]
					longitudenal connective	[66]
					Oesophageal commissure	[25, 81]
					Oesophageal commissure	[114]
					Schenkel des Mundringes/Nervenringes [German]	[76]
					Schlundkommissur [German]	[27]
					Schlundkonnektiv [German]	[28–31, 53, 74, 110]
					Stomodeal connective	[43]
Deutocerebral neuropil*	–	DN	11a, b, 12a–c, 13 and Additional file 5: Fig. S5b	Midline-spanning neurite region, which is located posterior to **→posterior dorsal neuropil** and appears as bump-like structure on posterior surface of brain neuropils; it is assigned to **→deutocerebrum**, as it receives fibres from neurons associated with **→jaw nerves** [15]	–	–
Deutocerebrum	see e.g. [15, 30, 48, 69]	DE	13 and Additional file 1: Fig. S1b, c	Part of **→brain** associated with **→jaw nerves** and belonging to second head segment; it consists of cell body rind and neuropil and is located posterior to **→protocerebrum**; it contains **→deutocerebral neuropil** as its only neuropil region	Frequently used term; synonyms not listed, as deutocerebral borders are ambiguous and inconsistent throughout the literature	–
Dorsal commissures*	–	DC	1d	Serially repeated bundles of neurites connecting **→ventral nerve cords**; each dorsal commissure projects laterally and proceeds dorsally, where they connect ventral nerve cords of both hemispheres; dorsal commissures link **→dorsolateral trunk nerves** and **→heart nerve**	Dorsal nerv	[43]
					Interpedalnerv [German]	[51]
					Interpedal nerve	[43]
					Interpedalnerv + Postpedalnerv + Präpedalnerv [German]	[29, 31, 51]
					Interpedal nerve + postpedal nerve + prepedal nerve	[56]
					Lateral commissure	[90]
					Lateral nerve	[82]
					Laterodorsal nerve	[95]
					motorischer Randnerv + Ringenerv + Postpedaler Nerv 1 und 2 [German]	[28]
					Nerve to segment	[91]
					Peripheral nerve	[41]
					Ring commissures	[14, 17–19, 37–40, 48, 68, 69, 116]
					Ringkommissur [German]	[111]
Dorsolateral trunk nerve*	–	DTN	–	Anteroposterior axon bundle proceeding in each dorsolateral region of trunk; it is crossed by and probably associated with numerous **→dorsal commissures**; its anterior and posterior extents are unknown	Dorsolateral bundle	[65]
					Dorsolateral nerve	[37, 48, 69]
					Dorsolateralnerv [German]	[111]
					Lateral bundle of nerve fibres	[117]
Fascicles of globuli cells*	–	FBC	2, 3, 6a, 7a–c, 11a, c, d, 13; arrows in 6a and Additional file 5: Fig. S5b; arrows in Fig. S2b	Fascicle-like, anteriormost neurite subunit of **→mushroom body** receiving fibres from **→globuli cells**; digitiform branches converge to four major branches, which then join **→pedunculus**; we refrain from using the term calyx because mushroom body fascicles of onychophorans apparently lack synaptic terminals and differ substantially in their morphology from calyces described for insects [118, 119]	Calyx	[17, 32, 33, 42, 48, 54]
					Globulibündel [German]	[27]
					Neurite der Globulizellen [German]	[31]
					masse médullaire [French]	[26]
					Stalks	[32]
					Stiele [German]	[51]
					Stiele der Globulizellen [German]	[31]
					Stielkomplex [German]	[30]
Frontal body*	–	FB	2, 3, 4a, 5c–e, 11a, b, 13 and Additional file 5: Fig. S5a, b	Peanut shaped, anteriormost midline neuropil located anterodorsally within **→protocerebrum** anterior to **→central body**; it contains prominent anterior and posterior strata with relatively high numbers of synapses	Anterior brain neuropil	[48, 69]
					Anterior neuropil	[38]
					Bridge	[32, 54, 62, 65]
					bourrelet médullaire antérieur [French]	[26]
					Dorsal superior protocerebrum	[33]
					Frontal brain neuropil	[55]
					Frontal brain neuropil	[17]
					Protocerebralbrücke [German]	[30, 53]
Globuli cells	[33, 42, 48]	GC	arrowheads in Additional file 2: S2a, c	Dense accumulation of somata within anterior cell body rind at anterior border of **→brain**; their axons innervate corresponding **→fascicles of globuli cells** of **→mushroom body**	Anterior cell masses	[56]
					Globule cells	[32]
					Globuli	[43, 56]
					Globuli [German]	[28, 50, 51, 53, 110]
					Globuligruppen [German]	[31]
					Globulipartie [German]	[27]
					Globulizellen [German]	[30]
					masse ganglionnaire antérieur [French]	[26]
Heart nerve	[14, 17, 37, 38, 68, 69]	HAN	–	Anteroposterior, dorsomedian axon bundle associated with dorsal wall of heart within trunk; it is linked with **→dorsolateral trunk nerves** and **→ventral nerve cords** via **→ dorsal commissures**; its anterior and posterior extents are unknown	Dorsal bundle of nerve fibres	[117]
					Dorsal nerve	[48]
					Dorsal nerve bundle	[65]
					Median cardiac nerve	[120]
					Herznerv [German]	[111]
Hypocerebral organ	[15, 34, 38–40, 48, 57, 61, 69–71, 97, 121]	HO	1c, 4c and Additional file 1: Fig. S1a	Vesicle-like structure attached to ventral surface of each brain hemisphere; its function is unknown, although it has been suggested to play endocrine role (e.g. [97, 104, 122])	Anhang des Kopfganglions [German]	[112]
					Appendage of the ventral surface of the brain	[25]
					Brain appendage	[60]
					Cerebral grooves	[123]
					Hypocerebral gland	[98]
					Hypocerebralorgan [German]	[50, 79, 111]
					Infracerebral organ	[34, 56, 63, 124–126]
					Infracerebral vesicle	[126]
					Infracerebral organ [Russian: инфра-церебральный орган]	[127]
					Infracerebralorgan [German]	[28, 50, 53]
					Infrazerebralorgan [German]	[51]
					kleiner Körper [German]	[76]
					organe ventral [French]	[26]
					organ ventraux du cerveau + ganglion intermediare [French]	[128]
					organe infracérébral [French]	[104, 122, 129]
					organe infra-cérébral [French]	[130]
					pear-shaped body	[43]
					spherule inférieure	[77]
					spherule infra-cervicale [French]	[131]
					Ventral appendage	[25, 81, 82]
					Ventral appendage of the brain	[28]
					Ventral body	[114]
					ventraler Gehirnanhang [German]	[91]
					Ventral lobe	[13, 34, 56]
					Ventral organ	[34, 43, 60]
					Ventralorgan [German]	[30, 52, 53, 73, 80, 108, 110, 132]
					Ventral protuberance of brain	[25, 81]
Inner lamina*	–	CB-IL	2, 3, 4c, 5a, c–e and 13	Neurite network in central part of **→central body** surrounded by **→outer lamina**; it shows inverted anteroposterior arrangement in **→anterior division** and **→posterior division** and encloses ventrally **→auxiliary neuropils**	–	–
Jaw nerve	[15, 39, 40, 48, 67–69]	JAN	1d, 2, 12a–c, 13 and Additional file 5: Fig. S5a–d	Axon bundle innervating each jaw (i.e. specialised limb of 2nd head segment) and associated with **→deutocerebrum**; it projects laterally at transition between **→brain** and **→circumpharyngeal nerve cord**, then finally bifurcates and runs ventrally to enter jaw	Jaw retractor muscle nerve	[95]
					Kiefernerv [German]	[28]
					Mandibelnerv [German]	[27, 31, 53, 74, 110]
					Mandibularnerv [German]	[28, 51]
					Mandibular nerve	[56]
					nerf des muscles mandibulaires [French]	[77]
					nerf mandibulaire [French]	[26]
					Nerve 11	[13]
					Nerve of feeding claw	[61]
					Nerve of the jaw	[25, 43, 81, 82, 126]
					Nerve to jaw	[25, 81, 82, 114]
					Nerve to jaw muscle	[91]
					Nervus mandibulairs [Latin]	[31, 53, 79]
Leg nerves	[3, 14, 17–19, 37–39, 41, 48, 68, 69, 116, 133, 134]	LEN	1c and d	Axon bundles innervating trunk limbs; each leg is supplied by anterior and posterior leg nerves that originate laterally from **→ventral nerve cord** and are segmental structures; associated somata are located on opposite side of nerve cord to bases of leg nerves [18]	Extremitätennerv [German]	[53, 79]
					Fußnerv [German]	[28, 29, 51, 135]
					nerf de patte [French]	[77]
					Nerve of walking leg	[40]
					Nerve to feet	[25, 81, 114, 115]
					Pedal nerve	[25, 43, 56, 82]
					Pedalnerv [German]	[29, 31]
Lip nerve 1*	–	LIN 1	1c, d and 2	Axon bundle innervating one of two anteriormost lip papillae; it originates at dorsal side of **→brain**, proceeds anteriorly, bends ventrally anterior to brain and finally innervates lip papilla; its associated somata are located within posterior **→protocerebrum** [40]	Anterior labial nerve	[56]
					First pair of lip papillae nerve	[48]
					hinterer Nerv des Mandibularsomits [German]	[28, 51]
					Lateral dorsal nerve	[43]
					Lip papillae nerve 1	[39, 40, 48, 69]
					nerf sympathique allant au papille du plafond buccal [French]	[77]
					nerve 7	[13]
					Nervus labialis anterior [Latin]	[30, 53]
					oral nerve	[38]
Lip nerve 2*	–	LIN 2	1d, 2, 12a–c, 13 and Additional file 5: Fig. S5c	Axon bundle innervating lateral lip papillae; it originates ventrolaterally from **→protocerebrum** and proceeds ventrally to innervate lip papillae 2–5 of corresponding hemisphere (in *E. rowelli*); it is exclusively sensory [40]	Labialnerv [German]	[28, 51]
					Labralnerv + oraler Ringnerv [German]	[74]
					Labral nerve + oral ring nerve	[91]
					Lateral labial nerve	[56]
					Lip papillae nerve 2	[39, 40, 48, 69]
					Lippennerv [German]	[28]
					Mundlippennerv [German]	[27, 31, 110]
					nerf labial [French]	[77]
					Nerve 8	[13]
					Nerve to circumoral fold	[43]
					Nervus labialis lateralis [Latin]	[30, 53]
					Oral nerve	[38]
					Second pair of lip papillae nerve	[48]
Lip nerve 3*	–	LIN 3	12c and 13	Axon bundle innervating posterior lip papillae; it originates from **→circumpharyngeal nerve cord** anterior to **→slime papilla nerve**s, proceeds anterior and innervates lip papillae 2–8 (8^th^ is unpaired in *E. rowelli*); its associated somata are located in **→deutocerebrum** and along circumpharyngeal nerve cord [40]	Lip papillae nerve 3	[39, 40, 48, 69]
					nerf de parois périlabiales [French]	[77]
					Nerve 12	[13]
					Nerve leading tot o the mouth lips	[68]
					Oral nerve	[38]
					Third pair of lip papillae nerve	[48]
Lobes (of the mushroom body)	[17, 33, 38, 42, 48, 86]	LO	2, 3, 4b, c, 6a, c, 8a, 13 and Additional file 3: Fig. S3a	Stipe of **→mushroom body** associated with **→pedunculus**; lobes are composed of four subcompartments including δ-lobe, λ-lobe, μ-lobe, and v-lobe	Columns	[32]
					Gehirntrabekel [German]	[27, 28, 30, 31]
					Gehirn-Trabekel [German]	[51]
					lames ventrales [French]	[26]
					Peduncle	[43]
					Stalk	[32, 54, 65]
					Trabeculae [Latin]	[53, 110]
					Trabekel [German]	[28]
					ventral lamellae	[90]
Macroglomerulus*	–	MG	4a, 8a, c and 13	Largest spherical subunit of **→olfactory lobe**; it is medianmost **→olfactory glomerulus** located in posterior part of olfactory lobe	Large glomerulus	[32]
Median lobe*	–	ML	11d, 12a, c and Additional file 5: Fig. S5a–d	Neuropil in the anterior ventral median brain; it emanates from **→subantennal neuropil** as a protrusion with tapered tip and receives fibres from **→olfactory fascicles 1–3**	–	–
Microglomeruli*; singular: microglomerulus	–	MIG	2, 3, 8a, b, e, f, 13 and Additional file 4: Fig. S4c, d	Diminutive, point-like accumulations of neurite terminals situated anterior to **→olfactory lobes**; they are embedded within **→subantennal neuropil**; synapsin staining reveals presence of multiple synaptic sites in each microglomerulus	–	–
Mushroom body	[13, 17, 32, 33, 38, 39, 45, 48, 54, 55, 57, 68–70, 108, 111, 112]	MB	2, 3, 4b, c, 6a–c and 13	Ventralmost neuropil of **→brain** associated with **→subantennal neuropil** and consisting of **→fascicles of globuli cells**, **→pedunculus** and four **→lobes**; fascicles receives fibres from **→globuli cells**; it is linked via **→ ponticulus** to **→central body**	corpus pedunculatum [Latin]	[30–32, 43, 56, 65, 68, 96]
					Pilzkörper [German]	[111]
Nephridial nerves	[14, 48, 69]	NEN	–	Axon bundle innervating nephridia; it arises from **→ventral nerve cord**, follows proximally specific **→ventral commissure**, and finally turns back distally to pass beneath ventral nerve cord and to join nephridial duct; nephridial nerves show bilateral and segmental arrangement [14]	–	–
Olfactory fascicles 1–4*	–	OLF	2, 3, 4a, 8a, b and 13	Neurite bundles emanating from **→olfactory lobes**, olfactory fascicles 1–3 circumvent **→mushroom body** and join **→median lobe** of brain neuropil region of ipsilateral brain hemisphere, whereas olfactory fascicle 4 connects to v-lobe and δ-lobe of mushroom body	faisceau de fibrilles [French]	[26]
					Fasciluli [Latin]	[110]
					fasciculi glomeruli [Latin]	[27]
					Fasciculi glomeruli [Latin]	[28]
					Projections	[33]
					Verbindungen [German]	[31, 53, 110]
Olfactory glomeruli; singular: olfactory glomerulus	[33, 38, 42]	OLG	2, 3, 4a, 5a–d, f, 13 and Additional file 4: Fig. S4a, b	Synaptic complexes, ball- or knot-like subunits of **→olfactory lobe** containing terminals of olfactory receptors with numerous other synaptic sites; most olfactory glomeruli are of similar size except for **→macroglomerulus**	Antennal glomeruli	[15, 17, 32, 34, 40, 42, 43, 48, 54, 65, 66, 68, 69]
					Antennalglomeruli [German]	[30, 31, 50, 53, 110, 111]
					Anterior appendage glomeruli	[13]
					glomérules olfactifs [French]	[26]
					Glomeruli	[33, 42, 51]
					Glomeruli [German]	[27, 28, 51]
					Glomeruli of the anterior appendage	[13]
Olfactory lobe	[33, 38, 42, 48, 57, 69, 71, 72, 96]	OLL	2, 6b, 8d, 11c and Additional file 2: S2b and Additional file 3: S3a	Entirety of **→olfactory glomeruli** of one brain hemisphere located anterolateral within **→brain** and embedded in **→antennal neuropil** and **→subantennal neuropil**; it is connected with **→median lobe** of brain neuropils via **→ olfactory fascicles 1–3** and with δ-lobe and v-lobe of **→mushroom body** via **→ olfactory fascicle 4**	antennal lobe	[17, 48]
					Glomerulargebiet [German]	[30]
					Glomerulipartie [German]	[28]
					Glomeruli-Partie [German]	[51]
					Glomeruli-Teil [German]	[51]
					Glomerulusteil [German]	[27]
					lobe olfactif [French]	[26]
					Olfactory neuropil	[57]
optic neuropil	[17, 38, 48, 55, 68]	ONP	2, 3, 4c, 10a–e and 13	Neuropil associated with eye and consisting of cup-shaped extracerebral part and tract-like intracerebral part, both of which consistently exhibit anti-synapsin immunoreactivity; it bifurcates into anterior and posterior branches; while anterior branch passes between **→antennal neuropil** and **→subantennal neuropil** to anteromedian position and vanishes in anti-acetylated α-tubulin and anti-synapsin stained brains (present study), posterior part passes through antennal neuropil and fuses with posterior stratum of **→posterior division** of **→central body**	Augennerv [German]	[28, 51]
					Augen-Nerv [German]	[51]
					Eye nerve [Russian: нерв глаза]	[101]
					lobe optique [French]	[26]
					nerf optique [French]	[26, 99, 101, 102, 109]
					Nerve 5	[13]
					nervus opticus [Latinised from Greek]	[30, 31, 73]
					Optic nerve	[25, 33, 40, 60, 81–83]
					Opticus [Greek]	[27, 53, 110]
					Optic ganglion	[25, 32, 65, 81, 83]
					Optic lobe	[43]
					Optic neuropils 1 and 2	[33, 42, 45, 75]
					Optic tract	[17, 32, 33, 38, 45, 47, 48, 54, 65, 69, 75, 136]
					pédicule optique [French]	[26]
					Sehmasse [German]	[30, 31]
					Sehnerv [German]	[31, 73]
					Tractus opticus [Latinised from Greek]	[30, 31, 52, 53, 79]
					Visual neuropil	[69]
					Visual neuropils 1 and 2	[33]
Outer lamina*	–	CB-OL	2, 3, 4c, 5a, c–e and 13	Neurite network and peripheral part of **→central body** surrounding **→inner lamina**; it shows inverted anteroposterior arrangement in **→anterior division** and **→posterior division**	–	–
Pedunculus; plural: pedunculi	[33]	PED	3, 4a, 5a, 7a–c, 13 and Additional file 2: Fig. S2b	Subunit of **→mushroom body** connecting **→fascicles of globuli cells** and **→lobes**; it gives rise to **→ponticulus** from its dorsomedian side	–	–
Pharyngeal nerve 1*	[39, 69]	PHN 1	1, 2, 11a–d, 12a, b, 13 and Additional file 5: Fig. S5c	Axon bundle associated with **→deutocerebrum** and innervating pharynx; it emerges posteriorly at indentation/fusion point of both brain hemispheres; it first proceeds anteriorly, then turns posteriorly and joins dorsolateral wall of pharynx; it extends to posterior end of pharynx, where pharyngeal nerves of both sides merge, forming loop; they are supplied by somata of serotonergic neurons distributed along pharyngeal wall [85] and are thus medullary structures	Eingeweidenerv [German]	[31]
					Frenal nerve	[56, 91]
					Frenalnerv [German]	[74]
					nerf viscerale [French]	[26]
					Nerve	[13]
					Nervus recurrens [Latin]	[28, 51–53]
					Nervus stomodeales [Latin]	[53]
					Nervus stomodealis [Latin]	[31]
					Paired stomodeal nerve	[56]
					Pharyngeal loop nerve	[48, 69]
					Stomatogastricus [Latin]	[27, 53, 110]
					Stomodaeal nerve	[43, 56]
					Sympathetic nerve	[25, 81, 82, 114]
Pharyngeal nerve 2*	[39, 69]	PHN 2		Diminutive axon bundle innervating pharynx; it originates from median part of **→circumpharyngeal nerve cord** anterior to **→subpharyngeal commissure** and innervates lateroventral wall of pharynx; most somata associated with this nerve are located in **→deutocerebrum** and only few in circumpharyngeal nerve cord [39]	hinterer Nerv des Mandilbularsomits [German]	[28]
					Stomodealnerv [German]	[74]
					Stomodeal nerve	[91]
Ponticulus*; plural ponticuli	–	PNT	2, 3, 4a, b, 6a, 7a–c and 13	From Latin *pontis* (=bridge); ponticulus connects **→pedunculus** of **→mushroom body** with **→outer lamina** of **→central body**; it shows neuropil-like organisation (with synapses) near pedunculus, but progressively turns into tract (without synapses) towards central body	Brücke [German]	[27]
					pédoncule [French]	[26]
					Peduncle	[90]
					Pedunculus [Latin]	[27, 28, 30, 31, 51, 53, 110]
					pedunculus	[32, 48, 54, 65, 69]
Posterior division (of central body)	[38]	CBP	2, 3, 5a, b and 13	Posterior subunit of **→central body**, which contains looping neurites forming **→outer lamina** and **→inner lamina**; **→anterior division** and **→posterior division** fuse laterally at dorsal part of central body; posterior division is associated with additional posterior stratum, from which it is separated by a discontinuous layer of somata and which is connected to posterior branches of **→optic neuropils**	hintere Lamelle des gestreiften Körpers [German]	[28]
					hinterer Zentralkörperteil [German]	[27]
					lame postérieure du bourrelet dorsal [French]	[26]
					Lamina posterior [Latin]	[32, 54, 65]
					Lamina posterior des Zentralkörpers [Latin + German]	[125]
					Posterior lamina	[17, 48, 69]
Posterior dorsal neuropil*	–	PDN	2, 4b, c, 5b–e, 11a, b, 12a–c, 13 and Additional file 5: Fig. S5b	Midline-spanning neurite region, which is part of brain neuropils; it is located in posterodorsal part of **→protocerebrum** posterior to **→central body** and anterior to **→deutocerebral neuropil**; it merges ventrally with **→posterior ventral neuropil**	Antennalkommissur [German]	[53, 110]
					Antennal commissure	[62]
					Central brain neuropil	[15, 17, 37, 48, 55, 59, 69, 70, 72, 98]
					Central commissural mass	[32, 54]
					Central fibrous mass	[56]
					Central lobe	[25, 81]
					Central neuropil	[13, 17, 32, 34, 38, 47, 48, 56, 59, 65, 69, 86]
					Central protocerebral commissure	[57]
					kommissurale dorsale Fasermasse [German]	[27]
					Median lobe	[25]
					obere Schlundkommissur [German]	[108]
					protocerebral commissure	[57]
					Zentralfasermasse [German]	[51]
					zentrale Kommissuren-Masse [German]	[51]
Posterior ventral neuropil*	–	PVN	2, 4b, 7a', c’, 11c, d, 12a–c, 13 and Additional file 3: Fig. S3d and Additional file 5: Fig. S5c	Midline-spanning neuropil of brain neuropil; it is located posteroventrally within **→protocerebrum** dorsal to **→mushroom body** and consists of neurite fibres emanating from **→subantennal neuropil**	Central brain neuropil	[15, 48, 55, 59, 70]
					Central fibrous mass	[56]
					Central lobe	[25, 81, 82]
					Central neuropil	[13, 17, 32, 34, 37, 38, 48, 55, 56, 59, 65, 69, 70, 86, 98]
					Median lobe	[25]
					Zentralfasermasse [German]	[51]
Protocerebrum	[15, 30, 48, 69]	PR	13 and Additional file 1: Fig. S1b	Part of **→brain** associated with **→antennal nerve cord** and belonging to first head segment; it is located anterior to **→deutocerebrum** and consists of cell body rind and following neuropils: **→frontal body**, **→central body**, **→mushroom bodies**, **→olfactory lobes, →optic neuropils**, **→ microglomeruli** and large unitary neuropil complex comprised of **→antennal neuropils**, **→subantennal neuropils**, **→median lobes**, **→central neuropil**, **→posterior dorsal neuropil**, **→posterior ventral neuropil** and **→ventral horns**	Frequently used term; synonyms not listed, as protocerebral borders are ambiguous and inconsistent throughout the literature	–
Salivary gland nerve*	–	SGN	13	Axon bundle supplying salivary gland; it emanates from **→ventral nerve cord** posterior to **→slime papilla nerve**, then turns posteriorly and innervates salivary gland [13]	Nerve 12	[13]
Slime papilla nerve	[15, 38–40, 48, 59, 66, 68, 69]	SPN	–	Paired axon bundle innervating each slime papilla (i.e. specialised limb of 3rd head segment); anterior and posterior slime papilla nerves emanate between **→lip nerve 3** and **→salivary gland nerve** at junction of **→circumpharyngeal nerve cord** and **→ventral nerve cord** and then turn anteriorly to enter slime papilla; associated somata are located within nerve cord opposite to bases of anterior and posterior slime papilla nerves [15]	nerf de tentacule buccal [French]	[77]
					Nerve 12	[13]
					Nerve of oral papilla	[43, 82]
					Nerve to oral papilla	[25, 81, 114, 115]
					Nervus papillaris [Latin]	[79]
					Papillar nerve	[61]
					Papillarnerv [German]	[28, 51]
					Papillennerv [German]	[74]
					Stomodeal commissure	[91]
Subantennal neuropil*	–	SAN	4a–c, 11b, c, 12a–c, 13 and Additional file 5: Fig. S5a, c, d	Neuropil, which situated laterally in each brain hemisphere; it is located ventral to **→antennal neuropil** and fuses posteriorly with **→posterior ventral neuropil**; it encompasses **→microglomeruli**, part of **→mushroom bodies** and **→olfactory lobes** and gives rise to **→brain commissure 3**	crête dorso-laterale [French]	[26]
					Lateral brain neuropil	[17, 48, 69]
					Postglomerular lateral neuropil	[13]
					Subantennalstrang [German]	[31, 51, 53, 110]
					subantennal lobe	[13, 32, 33, 54, 56, 62, 65]
					Subantennal tract	[32, 56]
					Subantennaltrakt [German]	[27]
					Subantennary tract	[56]
					Tractus subantennalis [Latin]	[27, 30, 31]
					Ventro-lateral horn	[25, 81]
Subpharyngeal commissure*	–	SPC	2, 12a–c, 13 and Additional file 1: Fig. S1a, c and Additional file 5: Fig. S5a, c, d	Neurite bundle, which is anteriormost **→ventral commissure**; sole commissure associated with **→circumpharyngeal nerve cords**	1. Kommissur [German]	[52]
					Commissural cord	[99]
					Commissure 1	[25, 82]
					commissure sous-œsophagienne [French]	[77]
					First ventral commissure	[43]
					Kommissur des Mandibularsomits [German]	[28, 51]
					Postoral commissure 1	[39]
					premiere commissure anterieure [French]	[102]
					Schlundkommissur [German]	[73]
					Sub-oesophageal commissure	[60]
					Tritocerebralkommissur [German]	[74]
					untere Schlundkommissur [German]	[80]
Suprarectal commissure*	–	SRC	1a, b	Posteriormost commissure linking **→ventral nerve cords** from both sides and forming loop above hindgut	Anal commissure	[82]
					Posterior dorsal commissure	[25, 81]
					Posterior-most commissure	[48]
Tongue nerve	[38]	TON		Axon bundle innervating tongue; it originates medial to **→hypocerebral organ** and proceeds into tongue [13]	Nerve 6	[13]
Ventral commissure	[43, 63]	VC	1c, d, 13 and Additional file 1: Fig. S1b and Additional file 5: Fig. S5b	Serially repeated median bundle of neurites connecting **→ventral nerve cords** from both body sides; anteriormost ventral commissure, which links **→circumpharyngeal nerve cords**, is specified as **→subpharyngeal commissure**	Commissur [German]	[31, 73, 76]
					commissura transversae [Portuguese]	[78]
					Commissural axons	[66]
					Commissure	[25, 33, 60, 81, 82, 102, 105, 114, 115]
					commissure [Russian: коммиссуры]	[101]
					commissure ventral [French]	[77]
					Communcationsnerven [German]	[76]
					cross commissure	[90]
					Kommissur [German]	[27, 29, 50, 74, 79]
					Median commissure	[3, 14, 17–19, 37, 39, 41, 48, 68, 69, 116, 121]
					Nerve commissure	[43]
					Postoral commissure	[39, 69]
					Tract-like commissure	[134]
					Transversal commissure	[100]
					Transverse commissure	[89]
					Transverse cord	[115]
					Quercommissur [German]	[112]
					Querkommissuren [German]	[28]
					Quernerven [German]	[76]
Ventral horn*	–	VH	4c, 11c, d and 12a–c	Cone-shaped neuropil; it emanates medially from **→posterior ventral neuropil** and extends anterior to level of **→median lobes**	–	–
Ventral nerve cord	[3, 13–15, 17–19, 25, 32, 34, 38–43, 48, 54, 56, 59, 61–63, 65, 67–72, 82, 83, 86, 90, 91, 93–96, 98, 100, 115, 116, 121, 137, 138]	VNC	1a–d, 13 and Additional file 1: Fig. S1c	Ventrolateral medullary nerve cord of trunk, which consists of peripheral cell body rind and central neuropil and anteriorly joins **→circumpharyngeal nerve**; ventral nerve cords of both sides are connected medially via **→ ventral commissures**, laterally via **→ dorsal commissures** and posteriorly via **→ suprarectal commissure**; each ventral nerve cord gives rise to lateral segmental **→leg nerves** at basis of each leg and to median segmental **→nephridial nerves**	Bauchkette [German]	[28, 29]
					Bauchmark [German]	[52, 53, 79, 110]
					Bauchnervenstrang [German]	[73]
					Bauchstrang [German]	[31, 51, 76, 113]
					cadeia nervosa ventral [Portuguese]	[78]
					chaîne ventrale [French]	[104]
					Connective	[33]
					cordon nerveux [French]	[77, 103]
					Längsnervenstrang [German]	[51, 80]
					Längsnervenstamm [German]	[28, 73, 80, 109]
					longitudinal connective	[66]
					Markstamm [German]	[139]
					Markstrang [German]	[111]
					Nervenstamm [German]	[29]
					Nervenstrang [German]	[76, 135, 140]
					Nerve-cord	[25, 60, 81, 99, 114, 141]
					Nerve-strand	[32, 54, 60]
					Nerve trunk	[89]
					Seitennerv [German]	[112]
					tronc ventral/nerveux [French]	[102]
					Ventral cord	[14, 25, 114]
					Ventral cord [Russian: брюшной ствол]	[101]
					Ventral nervous system	[33]

### General features of the nervous system

The central nervous system of *E. rowelli* consists of an anterodorsal →brain composed of a →protocerebrum and a →deutocerebrum, a pair of →circumpharyngeal nerve cords and the →ventral nerve cords (Fig. 1a, c, d and Additional file 1: Fig. S1a–c). The brain is associated ventrally with a pair of →hypocerebral organs (Fig. 1c). The ventral nerve cords run along each side of the body and are connected with each other via a posterior →suprarectal commissure looping above the hindgut (Fig. 1a, b). In addition, the nerve cords are linked along the body’s length by two types of serially arranged commissures: the →ventral commissures and the →dorsal commissures (Fig. 1c, d). The ventral commissures connect the two ventral nerve cords across the ventral midline, whereas the dorsal commissures project laterally, then cross one of the two →dorsolateral trunk nerves and finally connect the ventral nerve cords along the dorsal midline above the →heart nerve (cf. [14]). The dorsal commissures are missing in pedal regions, where instead a pair of segmental →leg nerves emanates from the ventral nerve cords on each body side (Fig. 1c, d). Each leg is supplied by an anterior and a posterior leg nerve. The same holds true for the slime papillae—specialised limbs of the third body segment—that are innervated by an anterior and a posterior →slime papilla nerve (Fig. 1d). The two slime papilla nerves are preceded by the →lip nerve 3 and succeeded by the →salivary gland nerve (cf. [13]). The circumpharyngeal nerve cords differ from the ventral nerve cords in that they are oriented dorsoventrally rather than anteroposteriorly and are not associated with commissures, except for a single, prominent →subpharyngeal commissure (Fig. 1a and Additional file 1: Fig. S1a, c).

Volume measurements reveal that the central nervous system of *E. rowelli* occupies ~ 3% of the total body volume, of which the brain constitutes only ~ 0.7% and the ventral nerve cords together with the circumpharyngeal nerve cords ~ 2.3% (Table 2). The central nervous system exhibits a clear subdivision into central neuropil and a peripheral cell body rind. Most somata are located in a ventral cell body rind and are largely lacking in most dorsal areas of the central nervous system, particularly in the ventral nerve cords (Fig. 1c, d and Additional file 1: Fig S1b, c and Additional file 6: Data S1, Additional file 7: Data S2).

**Table 2:** Volumes of brain and central nervous system in *E. rowelli* relative to total body volume based on SRμCT dataset

Structure	Relative volume (%)
Total body volume	100.00
Central nervous system	3.02
Brain	0.69
Ventral nerve cords and circumpharyngeal nerve cords	2.33

The brain consists of two ovoid, bilateral brain hemispheres fused along the midline (Fig. 1a, c and Additional file 1: Fig. S1b). Besides the posterior circumpharyngeal nerve cords, the most prominent bundles of neurites leaving the brain are the bilateral, anterior neuropils extending into the antennae, i.e. the →antennal nerve cords (Fig. 1a, c and Table 1). Among additional structures associated with the brain are a pair of anterolateral →optic neuropils supplying the eyes, a pair of ventral →tongue nerves projecting into the tongue (cf. [13]), and two pairs of lip nerves supplying the mouth (the mediodorsal →lip nerves 1 (Fig. 1c, d) and the posterolateral →lip nerves 2 (Fig. 12a–c)), whereas the lip nerves 3 are associated with the circumpharyngeal nerve cords (Fig. 12c; cf. [40]). In addition, the brain is associated with a pair of lateral →jaw nerves extending ventrally into the jaw musculature, and a pair of posterior pharyngeal nerves (→pharyngeal nerves 1) emerging at the indentation between the two brain hemispheres and entering the dorsolateral wall of the pharynx (Figs. 1c and 12 a, b). A second pair (→pharyngeal nerves 2) is associated with the circumpharyngeal nerve cords and enters the pharynx laterally (Table 1; cf. [39]).

### Organisation of the brain and associated structures

The brain of *E. rowelli* is comprised of an outer cell body rind and an inner neuropil region (Fig. 1c, d). The neuropil region constitutes 40–48% of the total brain volume, depending on the method used for fixation and further specimen treatment (Table 3). It is organised into distinct tracts, layers and neuropils (Figs. 2, 3, 4, 5, 6, 7, 8, 10, 11, 12 and Additional file 8: Data S3, Additional file 9: Data S4, Additional file 10: Data S5, Additional file 11: Data S6), which are characterised in the following.

**Table 3:** Volumes of cerebral structures in *E. rowelli* relative to total brain volume (TBV) based on SRμCT (entire head scan), and light (LM; serial sections of entire head) and confocal microscopy (CLSM; whole-mount preparations of brains)

Structure	Relative volume^**1**^ (%)			
	Ruthenium Red (SRμCT)	Azan (LM)	Synapsin (CLSM)	Acetylated α-tubulin (CLSM)
Brain (total)	100.00	100.00	100.00	100.00
Cell body rind	55.52	52.51	59.11	59.47
Brain neuropils	44.48	47.49	40.89	40.53
Brain commissures 1–3	–	–	–	0.60
Central body	–	3.02	2.447	2.50
Frontal body	–	–	3.277	4.13
Microglomeruli	–	–	0.027	0.01
Mushroom bodies	–	5.26	2.59^2^	4.23
Olfactory glomeruli	–	–	0.567	0.51
Olfactory fascicles	–	–	1.187	–
Optic neuropils	–	0.18	0.187	0.22
Ponticulus	–	0.11	–	0.09
Other brain neuropils	–	35.83	30.64	28.24

^1^Total volumes of structures from both brain hemispheres were estimated using corresponding numbers of voxels

^2^Excluding mushroom body’s fascicles of globuli cells

#### Frontal body

The →frontal body is a prominent anteriormost protocerebral midline neuropil occupying 3 to 4% of the total volume of the brain (Figs. 2, 3 and 5 c–e; Table 3). It borders anteriorly and dorsally the cell body rind and posteriorly the →central body but fuses laterally with the →antennal neuropil and ventrally with the →median lobes and →ventral horns (Figs. 4a, c, 11 and 12 and Additional file 8: Data S3, Additional file 9: Data S4, Additional file 10: Data S5, Additional file 11: Data S6). Parallel running fibres (or neurites) traversing the central body, connect the →ventral neuropil with the frontal body (Additional file 8: Data S3 and Additional files 9: Data S4). Anti-tubulin immunolabelling revealed an additional anteroventral connection to a branch associated with the antennal neuropil (Additional file 8: Data S3 and Additional files 9: Data S4). Although a histological subdivision of the frontal body into distinct layers is not evident, anti-synapsin labelling reveals highly immunoreactive strata in its posterodorsal and anteroventral regions (Fig. 5c, e; Table 1).

#### Central body

The →central body is the dorsalmost protocerebral midline neuropil occupying 2.4 to 3.0% of the total volume of the brain (Figs. 2, 3, and 5a–e; Table 3). It is an arcuate (lateral sides bent anteriorly), stratified structure situated posterior to the →frontal body. The central body can be subdivided into an →anterior division and a →posterior division. Anti-tubulin and anti-synapsin immunolabelling shows that both the anterior and posterior divisions are further stratified into an →inner lamina and an →outer lamina. Inner and outer laminae of both divisions are fused with each other at the lateral ends of the central body and therefore show an inverted anteroposterior arrangement in the anterior and posterior divisions (Fig. 5c–e). An additional posterior stratum is associated with the posterior division (Fig. 5c), from which it is delimited by a discontinuous layer of somata. Two relatively small, bilaterally symmetric, cylindrical →auxiliary neuropils are embedded ventrally into the inner lamina of the central body (Fig. 5a, d and e). Each auxiliary neuropil is linked via a bundle of neurites with the →antennal neuropil of the ipsilateral brain hemisphere (Additional file 8: Data S3 and Additional files 9: Data S4).

#### Mushroom bodies

The →mushroom body is a bilaterally paired neuropil located lateroventrally in the →protocerebrum (Figs. 2, 3 and 6 a–c). With 4.2–5.3% of the total brain volume, the mushroom bodies together constitute the largest individual neuropils of the brain (Table 3). They are embedded in the cell body rind of each brain hemisphere and adjoin dorsally the →subantennal neuropils (Fig. 4b). Each mushroom body is composed of three major subunits including (i) the →fascicles of globuli cells, (ii) the →pedunculus, and (iii) the four →lobes (Fig. 6a–c). The fascicle region comprises the anteriormost part of the mushroom body and lacks the presence of anti-synapsin labelled synaptic terminals (Fig. 2; Additional file 10: Data S5, Additional file 11: Data S6). It receives axon fibres from the →globuli cells located in the anterior cell body rind that converge to numerous digitiform branches. These smaller branches, in turn, anastomose to four larger branches that join the pedunculus (Fig. 6a–c and Additional file 2: Fig. S2a–c). The pedunculus gives rise posteriorly to four lobes that are sickle-shaped, laterally convex structures. Based on their position, we distinguish a dorsal (δ-lobe), lateral (λ-lobe), medial (μ-lobe) and a ventral lobe (v-lobe; Fig. 6a, c). While the δ-lobe and the v-lobe are stacked on top of each other, the μ-lobe and the λ-lobe encompass them medially and laterally, respectively. All four lobes are separated from each other by thin, discontinuous layers of somata (Fig. 4b, c). We were unable to determine whether these belong to glial cells. Immunoreactivity against synapsin is consistently stronger in the μ-lobe and weaker in the δ-lobe than in the v-lobe and the λ-lobe (Fig. 4b, c).

#### Ponticulus

The →ponticulus extends from the →pedunculus of the →mushroom body to the →outer lamina of the →central body (Fig. 6a). The ponticulus shows a mixed tract-like/neuropil-like organisation, as the anti-synapsin signal is high near the pedunculus but progressively decreases and finally disappears towards the central body (Fig. 4a, b), whereas acetylated α-tubulin is distributed along its entire length (Figs. 2, 3; Additional file 8: Data S3 and Additional file 9: Data S4). Moreover, synapsin is not distributed evenly but shows a granular arrangement (Fig. 4a). The ponticuli of both brain hemispheres constitute ~ 0.1% of the total brain volume (Table 3).

#### Brain commissures 1–3

The brain of *E. rowelli* exhibits three, individually identifiable branched commissures, identified by Azan-labelled sections and anti-tubulin immunostaining: the →brain commissures 1–3 (numbered from posterior to anterior), formed by contralaterally projecting bundles of axon fibres crossing the →protocerebrum (Figs. 2, 3 and 7a–c; Additional file 2: Fig. S2a–c). These prominent commissures occupy together ~ 0.6% of the total volume of the brain (Table 3). Two of them, the brain commissures 1 and 2, crosslink the →antennal neuropils of each brain hemisphere, whereas the brain commissure 3 connects the →subantennal neuropils of both brain hemispheres (Fig. 7a–c). None of these commissures are directly associated with the →mushroom bodies, from which they are spatially separated by fibres belonging to the →posterior ventral neuropil and subantennal neuropil (Fig. 7a–c and Additional file 3: Fig. S3a–f). The brain commissure 1 originates from the antennal neuropil, then passes through the space between the →pedunculus and the →ponticulus and follows the μ-lobe of the mushroom body to finally cross the midline of the brain (Fig. 7a). The brain commissure 2 fasciculates immediately after leaving the antennal neuropil into three branches (Fig. 7b). All three branches flank the posterior margin of the pedunculus and proceed further posteriorly above the →lobes of the mushroom body. At this point, the lateral branch terminates, whereas the other two branches merge and continue as a single commissure towards the contralateral brain hemisphere. The brain commissure 3 is situated anterior to the brain commissure 2 and connects the contralateral subantennal neuropils (Fig. 7c and Additional file 8: Data S3 and Additional files 9: Data S4). After leaving the subantennal neuropil, this commissure passes dorsal to the μ-lobe of the mushroom body and then splits into two branches that finally cross the posterior ventral neuropil to the contralateral side.

#### Antennal pathways

The **→**antennal nerve cords, which are associated with the protocerebral antennae [13, 15], enter the brain frontally and continue without any recognisable neuroanatomical border into the two →antennal neuropils (Fig. 11a, b) and the →olfactory lobes (Figs. 2, 3 and 8a–d; cf. [15]). Together with the →mushroom bodies, the olfactory lobes represent the ventralmost neuropils of the brain. Each olfactory lobe is an elongated, irregular assemblage of ~ 80 subunits, the →olfactory glomeruli, which are largely, albeit not exclusively, located in the periphery of the lobe (Fig. 8a–d). The olfactory glomeruli from both olfactory lobes constitute together ~ 0.5% of the total brain volume (Table 3). Each olfactory glomerulus represents a synaptic complex with numerous synaptic sites (Additional file 4: Fig. S4a, b). The olfactory glomeruli appear spherical, ellipsoid or ovoid in shape (Fig. 8a–d). They also vary in size, ranging in diameter from ~ 9 to 40 μm (average diameter 19 μm), with most glomeruli falling into the range of 10–25 μm (Fig. 9). The median posterior half of each olfactory lobe harbours a single, particularly large glomerulus, which measures 40 μm in diameter and is therefore referred to as the →macroglomerulus (Figs. 8a, c and 9). Volume measurements reveal that the macroglomerulus is ~ 7 times larger than a medium sized olfactory glomerulus (Table 4). Synapsin immunolabelling further revealed four →olfactory fascicles (1–4) associated with each olfactory lobe, three of which (1–3) are thinner (diameter 5.5 μm) than the fourth one (diameter 20.3 μm; Fig. 8a and b). While the olfactory fascicles 1–3 are associated with the anterior part of the olfactory lobe and join the ipsilateral →median lobe of the →brain (Additional file 10: Data S5 and Additional file 11: Data S6), the olfactory fascicle 4 originates further posteriorly and fuses with the v-lobe and the δ-lobe of the mushroom body (Fig. 8a, b and Additional file 10: Data S5 and Additional file 11: Data S6).

**Table 4:** Relative volumes of three glomerular neuropils in the brain of *E. rowelli* based on anti-acetylated α-tubulin immunolabelling

Structure	Relative volume (%)
Macroglomerulus	100.00
Microglomerulus	0.69
Olfactory glomerulus (medium sized)	14.34

#### Microglomeruli

We further identified microglomerular complexes in the anterolateral part of each brain hemisphere, where they are embedded in the →subantennal neuropil. Each complex consists of ~ 40 units, the →microglomeruli, which show a cup-shaped arrangement anterior to the →olfactory lobes and are revealed by both, anti-synapsin and anti-acetylated α-tubulin immunolabelling (Figs. 2, 3 and 8 a, b, e, f; Table 1). Each microglomerulus is spherical in shape and represents a complex of numerous, prominent synaptic sites, as revealed by anti-synapsin immunolabelling (Additional file 4: Fig. S4c and d). A single microglomerulus measures 4.5–6.5 μm in diameter and comprises only one-twentieth the volume of a mid-sized →olfactory glomerulus; all microglomeruli together constitute only 0.01–0.02% of the total brain volume (Tables 3 and 4 and Additional file 4: Fig. S4c and d).

#### Visual pathways

The →optic neuropil is comprised of extra- and intracerebral parts that label consistently with anti-synapsin (Fig. 10a). The extracerebral part appears as a cup-shaped structure within each of the two eyes and continues via a short, bottleneck-shaped connection into the brain (Figs. 2, 3 and 10 a and b). The intracerebral part of the optic neuropil then splits into an anterior and a posterior branch (Fig. 10a, b). The anterior branch projects anteromedially between the →antennal neuropil and the →subantennal neuropil, but could not be traced further in our datasets (Additional file 8: Data S3, Additional file 9: Data S4, Additional file 10: Data S5, Additional file 11: Data S6). The posterior branch crosses the →antennal neuropil and passes dorsally towards the →central body (Fig. 10a, d). It then splits into an anterior and a posterior bundle of fibres (Fig. 10a, e). The anterior bundle runs towards the →anterior division of the central body, passing it ventrally without entering before disappearing in the fibre meshwork of the →central neuropil (Fig. 10a, c). The posterior bundle proceeds towards the →posterior division of the central body and enters the associated posterior stratum, after which the anti-synapsin signal vanishes (Fig. 10a, e). The optic neuropil constitutes only ~ 0.2% of the total brain volume (Table 3).

#### Other brain regions

Although our methods allow for a clear distinction of various tracts, layers and neuropils in the →brain of *E. rowelli*, some large brain areas are difficult to characterise because of their comparatively homogenous appearance. These areas occupy about two-thirds of the entire brain neuropil and one-third of the total brain volume and appear as a relatively homogeneous and complex meshwork of fibres (Fig. 11a–d; Table 3). For descriptive purposes, we subdivided and colour-coded these brain neuropils into defined regions (Figs. 11a–d and 12b, c). We distinguish bilateral midline-spanning →central neuropil, →posterior dorsal neuropil, →posterior ventral neuropil and →deutocerebral neuropil as well as paired, separate →antennal neuropils, →subantennal neuropils, →median lobes and →ventral horns (Figs. 11a–d, 12a–c; Table 1).

The →antennal neuropil receives input from the →antennal nerve cord and is ventrally adjacent to the →subantennal neuropil (Figs. 12b, c). Posteriorly, it fuses with the →posterior dorsal neuropil, which occupies the posterodorsal region of the →protocerebrum. The subantennal neuropils give rise to a pair of horn-like median lobes and merge with the posterior ventral neuropil. The posterior ventral neuropil forms an arcuate structure, which gives rise to a pair of elongated, cone-shaped ventral horns that project anteriorly and align with the anterior border of the median lobes (Figs. 11b, c and 12c, d). A small, unitary central neuropil is located anterior to the posterior ventral neuropil and the posterior dorsal neuropil, beneath the central body (Figs. 11b and 12b, c).

The posterior dorsal neuropil is succeeded posteriorly by the deutocerebral neuropil, apparent as a bump-like structure in the posterior region of the brain (Figs. 11a and 12a, b). We assign this region to the →deutocerebrum (Table 1) because it receives fibres from neurons supplying the jaws that are specialised limbs of the second cephalic segment (cf. [15]).

The posterior dorsal and posterior ventral neuropils, which comprise the largest accumulations of fibres linking both brain hemispheres, finally continue posteriorly into the neuropils of the two →circumpharyngeal nerve cords, again without any visible neuroanatomical transition (Figs. 11a, b, 12a–c). The neuropils of the above described, unitary fibre meshwork give rise to all nerves associated with the brain. While the →lip nerves 1, the →lip nerves 2 and the →tongue nerves arise from the protocerebral part, the →pharyngeal nerves 1 and the →jaw nerves are associated with the deutocerebral neuropil.

## Discussion

**Fig. 13:**
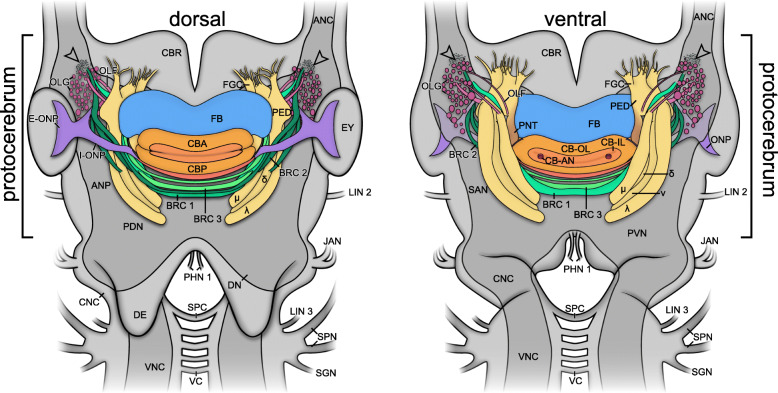
Simplified diagram illustrating major commissures, tracts, lobes and neuropils in the brain of *E. rowelli*. Characterised neuropils are depicted in colour; other brain neuropils are indicated in dark grey, cell body rind in light grey. Subregions of largely fused other brain neuropils, including deutocerebral neuropil, not shown. Arrowheads point to microglomeruli. ANC, antennal nerve cord; ANP, antennal neuropil; BRC 1, BRC 2, BRC 3, brain commissures 1 to 3; CBA, anterior division of central body; CB-AN, auxiliary neuropil; CB-IL, inner lamina of central body; CB-OL, outer lamina of central body; CBP, posterior division of central body; CBR, cell body rind; CNC, circumpharyngeal nerve cord; δ, dorsal or δ-lobe of mushroom body; DE, deutocerebrum; DN, deutocerebral neuropil; E-ONP, extracerebral part of optic neuropil; EY, eye; FB, frontal body; FGC, Fascicles of globuli cells; I-ONP, intracerebral part of optic neuropil; JAN, jaw nerve; λ, lateral or λ-lobe of mushroom body; LIN 2, LIN 3, lip nerves 2 and 3; μ, median or μ-lobe of mushroom body; OLF, olfactory fascicle; OLG, olfactory glomeruli; ONP, optic neuropil; PDN, posterior dorsal neuropil; PED, pedunculus; PHN 1, pharyngeal nerves 1; PNT, ponticulus; PVN, posterior ventral neuropil; SAN, subantennal neuropil; SGN, salivary gland nerve; SPC, subpharyngeal commissure; SPN, slime papilla nerves; v, ventral or v-lobe of mushroom body; VC, ventral commissure; VNC, ventral nerve cord

Our multiscale approach unveiled the organisation of the onychophoran brain at different hierarchical levels of detail (Fig. 13). While the analysis of SRμCT data and Azan-stained sections of entire heads did not require brain dissection and, thus, revealed the least distorted shape of the individual structures, anti-acetylated α-tubulin and anti-synapsin immunolabelling of dissected brains were most informative for demonstrating the intracerebral elements and their components. We used this information for reconstructing the architecture of the brain in *E. rowelli* and refining the neuroanatomical terminology for Onychophora (Table 1). The results help to clarify persisting controversies surrounding the identity and homology of specific structures and regions in the onychophoran brain.

### Anatomy and homology of mushroom bodies

Volume measurements revealed that the mushroom bodies are the largest individually identifiable cerebral neuropils in *E. rowelli*. Homonymous structures have been reported from other protostomes, including arthropods and some lophotrochozoans [142–150]. Despite the proposed common ancestry across protostomes (discussed in [53, 55]) and hypothesised homology with the vertebrate pallium [151], the homology of mushroom bodies has not been entirely settled even across arthropods [84].

This uncertainty might be due to considerable variation across arthropods. For example, the calyx is lacking in myriapods [147], paleopteran and some neopteran insects [118], and partially also in stomatopod crustaceans [152]. Moreover, a structure called the hemiellipsoid body—a potential homologue of the mushroom body calyces (Bellonci 1982 *apud* [153, 154]) or the mushroom body itself [155, 156]—has so far been identified in only three crustacean clades: Malacostraca [152, 157–160], and Remipedia [161], which both lack the mushroom body pedunculi and lobes, as well as Cephalocarida [162]. This suggests either an independent origin in these clades or an evolutionary loss in other crustaceans. In a study using an antibody (anti-DC0) against the catalytic subunit of protein kinase A [163, 164], which selectively labels the columnar neuropils in the mushroom bodies of insects (e.g. [165, 166]) and other arthropods [147, 152, 158, 159], Strausfeld et al. [148] provided evidence that the mushroom bodies, or at least the mushroom body calyces, generally occur in crustaceans. This suggests that these structures are homologous at least across arthropods [147].

Hence, the question arises whether mushroom bodies were present in the last common ancestor of panarthropods. While comparable structures have not been identified yet in tardigrades (e.g. [85, 167]), mushroom bodies (corpora pedunculata) were recognised early in onychophorans [27, 28, 30, 31]. As in arthropods, they are located in the protocerebrum, a homologous brain region in the two clades [15, 33, 42, 48, 54, 69, 168]. Moreover, they show a subdivision into a fascicle region, a pedunculus and four lobes (Fig. 13; see also [33]). The fascicle region is associated with characteristic globuli cells, which most likely correspond to the globuli cells that give rise to processes within the calyces of arthropods [23, 143].

However, as opposed to most insects and crustaceans, the globuli cells in onychophorans do not contribute to a cup-like structure called calyx [119]. As in calyx-less insects [118, 119], the globuli cells of onychophorans instead contribute to fibre fascicle that converge to the pedunculus. This resembles also the situation in the calyx-less mushroom bodies of myriapods, paleopterans and some neopterans, where globuli cells form neurite bundles that extend into the pedunculus [118, 147]. It is largely accepted, however, that lack of calyces has occurred independently in paleopterans and some neopterans within the Hexapoda lineage [118]. In chelicerates, mushroom bodies including calyces (or mushroom body heads) have been described for several species (e.g. [150, 169–172]). However, the structure of their calyces is substantially different from the typical insect/crustacean calyces [e.g. 169]. Considering that the mushroom body calyces, as defined for insects [119] and crustaceans, are absent in chelicerates, myriapods and onychophorans, one may conclude that this structure first emerged in the pancrustacean lineage [118] and that onychophoran mushroom bodies most likely resemble the ancestral condition within Panarthropoda.

Accordingly, positional and structural similarities currently speak in favour of the proposed homology of mushroom bodies in onychophorans and arthropods [32, 42, 54]. Even so, analysing the expression patterns of genes that are involved in learning [147] might assist in substantiating this hypothesis. Localising anti-DC0-immunoreactive centres in the onychophoran brain might further help to identify homologous regions, as shown in numerous comparative studies across pancrustaceans (e.g. [148]) and other arthropods [147].

Besides these similarities, our study also revealed differences and previously unnoticed details in the organisation of mushroom bodies in velvet worms. One of these details concerns the number of mushroom body lobes. While only three of them were reported previously ([26, 27, 31, 33, 42, 48, 80]; but see 32 for suggested existence of additional compartments), we identified four lobes (δ, λ, μ and ν) in each mushroom body of *E. rowelli* with three independent methods: Azan staining, anti-synapsin and anti-tubulin immunolabelling. Notably, while the μ-lobe exhibits the strongest anti-synapsin immunoreactivity, suggesting dense synaptic connectivity, the signal is nearly undetectable in the δ-lobe. Interestingly, the μ-lobe of *E. rowelli* also expresses high levels of arthropsin, an opsin (G protein-coupled receptor) with unknown function [86]. Immunolocalisation of pigment-dispersing factor neuropeptides, Er-PDF-I and Er-PDF-II, revealed a similar conspicuous pattern, with most fibres occurring in the μ-lobe [38]. This hints at a functional specialisation of the individual lobes of the mushroom body, which remains to be clarified, e.g. by gene knockouts combined with behavioural assays or electrophysiological recordings (cf. [55]).

Such experiments might also help to clarify the role of the onychophoran mushroom body as a whole, which is still unknown. Does this neuropil serve as an integrative centre for olfactory, visual and place learning and memory, with the additional role as a general behavioural control centre, as in insects [173–179]? Answers may emerge from expression studies of specific proteins, such as Leo and pCaMKII, that are present in paired lobed centres in the brain of representatives of all four major arthropod groups [147] and are required for learning and memory in the fruit fly *Drosophila melanogaster* [164, 180, 181].

Another contentious issue concerns the identity of the “accessory lobes”, also referred to as “accessory stalks” or “Nebentrabekel”, that have been repeatedly alleged to be associated with onychophoran mushroom bodies [30, 32, 33, 42, 48, 54, 69]. As in chelicerates, but in contrast to other arthropods, these structures are believed to link the mushroom body of both brain hemispheres across the midline. This feature has also been used to unite onychophorans with chelicerates, which exhibit a similar connection [42]. However, our 3D reconstructions clearly show that the corresponding heterolateral connections are associated with the antennal and subantennal neuropils (Fig. 13), but not with the mushroom bodies directly. We refer to these connections as brain commissures 1–3 (Table 1), as they do not seem to contain many synaptic sites and were only discernible with anti-acetylated α-tubulin immunolabelling. Since these commissures are not part of the onychophoran mushroom bodies, they cannot be used for justifying the sister group relationship of onychophorans and chelicerates [42, 182]. The connection between the mushroom bodies of both brain hemispheres in chelicerates might instead have evolved independently within their lineage.

### Controversies surrounding the frontal body

The frontal body comprises the second largest individually identifiable cerebral neuropil in *E. rowelli*, but has rarely been noted in onychophorans. Saint-Remy [26] initially described this midline neuropil in the brain of *Peripatopsis capensis* as “bourrelet médullaire antérieur” (i.e. anterior medullary ridge). Almost five decades later, Hanström [30] rediscovered it in *P. capensis*, *Kumbadjena occidentalis* and *Ooperipatellus insignis* and homologised it with the protocerebral bridge of crustaceans and insects. However, his descriptions are inconsistent, as differing regions are labelled as “Protocerebralbrücke” in his illustrations (cf. figs. 12 and 13 in [30]). Likewise, Schürmann [32, 54] identified a V-shaped midline neuropil anterior to the central body as “bridge” in *E. leuckartii*, a species closely related to *E. rowelli*, but at the same time he assigned the two divisions of the central body to it (cf. fig. 8.2C in Schürmann [32]). It is worth mentioning that Strausfeld et al. [33] recognised the corresponding region as “dorsal heterolateral neuropil” and “dorsal superior protocerebrum”, whereas Mayer [48] and Martin et al. [38] referred to it as “anterior neuropil”, although none of these studies provided a detailed description. Beyond this, we know of no further reference to the frontal body in onychophorans. This is astonishing, given its considerable size and conspicuous shape and position in the onychophoran brain (Fig. 13).

Our 3D reconstructions of the frontal body in *E. rowelli* match superficially Schürmann’s [32, 54] illustrations of the “bridge”. Although his schematised “bridge” is considerably smaller and widely separated from the central body, Schürmann [32, 54] correctly depicted its shape and superficial, anterior position relative to other brain neuropils. The issue of whether the frontal body of onychophorans is homologous with the protocerebral bridge of crustaceans and insects, as suggested by Hanström [30], is currently difficult to address due to limited information on its internal organisation. We have shown here that synaptic proteins are mostly accumulated in the anterior and posterior regions of the frontal body, indicating at least some degree of regionalisation and layering. Contrary to onychophorans, the protocerebral bridge of insects and certain crustaceans shows a clear subdivision into distinct columns, slices or glomeruli [183–187] that do not seem to correspond to the transverse layers found in *E. rowelli*. Furthermore, the onychophoran frontal body lacks a connection to the central body, which differs from the situation in insects, where the protocerebral bridge is clearly associated with the central body [183]. Although differences per se do not invalidate homology, given the lack of compelling evidence and the absence of the corresponding neuropil in chelicerates and myriapods, we regard the proposed homology [31, 32] of the frontal body of onychophorans with the protocerebral bridge of pancrustaceans as unlikely.

### Organisation of the central body revisited

The central body is the third largest individually identifiable neuropil in the brain of *E. rowelli* (Fig. 13). Our 3D reconstructions correspond well to previous descriptions of this midline neuropil [26, 27, 32, 33, 42, 48, 56, 69], except that we identified a lateral fusion of the bilobed structure at the ventral part of the central body. Anti-acetylated α-tubulin and anti-synapsin immunolabelling further revealed two prominent strata—the inner and the outer laminae—which show an inverted anteroposterior arrangement in the two divisions. The two laminae most likely correspond to the successive strata of looped axons and tangential fibres described by Strausfeld et al. [42], suggesting a complex wiring. We further detected a posterior stratum associated with the posterior division, which receives fibres from the optic neuropil, as well as a pair of auxiliary neuropils linked to the antennal neuropils. This indicates processing of visual input (from the eyes) and olfactory and/or mechanosensory information (from the antennae) in the central body.

Besides bilateral input from the optic neuropils, our findings confirm the existence of a thick fibrous connection, the ponticulus, which links each mushroom body to the central body in *E. rowelli*. This connection was first described as “pédoncule” by Saint-Remy [26] and since then is assumed to be associated with either the anterior division or the posterior division of the central body [26–28, 30–32, 54]. Our 3D reconstructions revealed a specific connection of each ponticulus to the posterior region of outer lamina of the central body. Interestingly, while the ventral part of the ponticulus contains numerous granular synaptic complexes, these are completely lacking in its dorsal part. The progressive dorsoventral increase of synaptic sites suggests a combined tract-like/neuropil-like organisation. Similar distributions of synapsin have been observed in the olfactory globular tract of crustaceans toward the olfactory globular tract neuropil [188–190] and along the mushroom body pedunculus of insects and spiders [191–193]. Nonetheless, the ponticuli, which directly link the mushroom bodies and central body, appear to be a unique feature of Onychophora. While direct connections between the mushroom bodies and central complex are conspicuously absent in insects [194, 195], recent connectome data from *Drosophila* revealed multiple indirect connections from mushroom body output neurons primarily to the fan-shaped body of the central complex [178, 179], a centre proposed to serve a role in spatial orientation and navigation [196–198]. The central body of insects is generally believed to be an integrating centre that processes information from both brain hemispheres and, together with the protocerebral bridge and noduli, controls goal-directed locomotion, including directional control of walking and flight, spatial visual memory, place learning, sky compass orientation and path integration (e.g. [183, 199, 200]). Behavioural experiments have demonstrated that velvet worms avoid light and are instead attracted by darkness [55, 201]. Given the complexity of their locomotor behaviour [202, 203], myoanatomy [19] and neuronal supply to individual legs [3, 18], it is conceivable that the central body of onychophorans might integrate visual and antennal information related to locomotion. A possible function in directional control of locomotion might explain the relatively large size of this midline brain neuropil (Fig. 13).

The central body of onychophorans has been homologised with that of arthropods [30, 149]. This was, however, not always the case, due to dissenting opinions on the homology of the arcuate body of chelicerates with the central body of other arthropods ([42]; discussed in [204]). Similarities in the organisation into multiple layers as well as its superficial position within the brain and direct connection to the visual system might indeed support homology of the onychophoran central body with the arcuate body of chelicerates [42]. Myriapods might play a key role in elucidating the evolution of midline neuropils across arthropods, as they show similarities to both chelicerates and pancrustaceans [84, 204–206]. While the central body of myriapods on the one hand shares to some extent a layered organisation with the arcuate body of chelicerates [205, 207], it is on the other hand embedded deeper within the protocerebral neuropil, as in pancrustaceans [161, 184, 186, 208]. Furthermore, to our knowledge, nothing is known about a potentially corresponding lobular midline neuropil in tardigrades (but see, e.g. [85, 167, 209] for an uncharacterised “central brain neuropil”). Taking all available information into account, we propose that the last common ancestor of Onychophora + Arthropoda most likely possessed a transverse, arcuate midline neuropil, which was composed of successive strata and had a superficial position in the protocerebrum (i.e. abutting the other protocerebral neuropils), since these features are found in both onychophorans and chelicerates [42]. Whether the individual strata in chelicerates correspond to the inner and outer laminae in onychophorans might be clarified by specific labelling using antisera against neurotransmitters and neuropeptides (e.g. [152, 207, 210, 211]).

### Novel microglomerular neuropils with uncertain role

Anti-acetylated α-tubulin and anti-synapsin immunolabelling of *E. rowelli* revealed miniscule cerebral complexes, the microglomeruli, that occupy only about 0.01% of the total brain volume and consist of ~ 40 individual nodules (Fig. 13). Microglomerular neuropils have been reported from different brain regions of distantly related arthropods (e.g. [193, 212–214]). In some insects, microglomeruli play a role in visual pathways associated with sky compasses [213, 215, 216]. Furthermore, microglomerular neuropils are associated with cells in the mushroom body calyces [212, 217–221] that are referred to as Kenyon cells [208]. A similar arrangement was found in crustaceans, in which microglomeruli are associated with the hemiellipsoid body [222–225], the potential homologue of the mushroom body [152, 225]. In chelicerates, microglomeruli might play a role as second-order visual neuropils, as they are associated with the secondary eyes in spiders [193, 226].

The association of microglomeruli with olfactory lobes has also been described for the accessory lobes of decapod crustaceans [190, 223] and the antennal lobes of distinct insect groups, including orthopterans and coleopterans [214, 227, 228]. The accessory lobes of decapods are second-order association centres located in the deutocerebrum and always occur in close proximity to the olfactory lobes. Within decapods, accessory lobes most likely evolved in the reptantian lineage [229]. Amongst others, these higher-order neuropils receive olfactory information from the olfactory lobes via interneurons [230, 231]. Similarly, microglomeruli are present in the antennal lobes of two distantly related representatives of coleopterans, the diving beetles and lady bugs [228]. Several coleopteran sub-groups also show a microglomerular compartmentation of glomeruli, as revealed by either immunolabelling for synapsin and/or tachykinin-related peptides [228].

We have shown here that microglomeruli are closely associated with olfactory lobes and antennal neuropils in the brain of *E. rowelli*, suggesting that they might be connected with olfactory pathways, as for certain microglomeruli of insects [214]. Despite the close association with the olfactory glomeruli, a potential role of microglomeruli in olfaction requires further exploration. An involvement in visual pathways, as described from several arthropod groups [193, 213, 215, 216, 232], seems unlikely, as we did not find signs of any connection to the visual system.

### Olfactory glomeruli: a remarkable case of homoplasy across panarthropods

Olfactory glomeruli are defined as dense neuropils, in which the olfactory receptor neurons terminate and form the first synapses of the olfactory pathway [20]. They occur in various metazoans and have been extensively studied in arthropods (e.g. [206, 227, 233, 234]). To our knowledge, comparable structures have not been recorded in tardigrades. As in arthropods, the olfactory glomeruli of onychophorans are arranged in clusters referred to as olfactory lobes [33, 42, 48, 57, 69]. They most likely represent the primary processing centres of olfactory stimuli, as they are supplied by neurites associated with the antennae [15, 124, 168]. While the olfactory lobes of onychophorans are located in the protocerebrum [31, 32], those of arthropods are situated either in the deutocerebrum, as in most mandibulates [206, 233], or exhibit great variability with respect to segmental identity, as in chelicerates [42]. For example, in solifuges, scorpions, amblypygids and pycnogonids, the olfactory lobes are associated with post-cerebral locomotory appendages that serve as tactile organs [235–237]. Solifuges possess in addition a glomerular neuropil in the tritocerebral brain segment connected to the pedipalps [42] and a further set of olfactory glomeruli occur in close proximity to the mushroom body calyces, at least in solifuges and amblypygids [150, 235]. Taken together, the variable position and different segmental identity do not support homology of olfactory lobes across panarthropods. The similar organisation of olfactory neuropils in distantly related groups might rather be due to functional constraints associated with olfactory processing [238, 239].

Although a recent study revealed that onychophorans might have lost some chemosensory gene families found in protostomes including arthropods [240], the prominent size of olfactory lobes in *E. rowelli* indicates that olfaction nevertheless plays an important role in these animals. While each olfactory lobe of different onychophoran species, including our study species, was previously thought to contain only about 30 glomeruli arranged in two clusters [31–33], our 3D reconstructions revealed that each lobe of *E. rowelli* constitutes a single, elongated cluster of ~ 80 glomeruli that differ in size and exhibit a stereotypic arrangement (Fig. 13). The high number of ~ 80 olfactory glomeruli per body side of different sizes and shapes in *E. rowelli* imply a prominent role of olfactory processing in onychophorans, which might be correlated with predatory behaviour and nocturnal lifestyle combined with low-resolution vision and chemical communication in these animals [70, 201, 241, 242].

We have shown here that each olfactory lobe of *E. rowelli* contains a median macroglomerulus (∅ 40 μm), whereas most other glomeruli do not exceed half this diameter. This variation in size, already pointed out by Schürmann [32], might indicate functional specialisation of the individual glomeruli. Similar macroglomerular complexes occur in various insects, in which they process pheromone signals, including those from trail pheromones [243–245]. The single macroglomerulus in each brain hemisphere of *E. rowelli* might have a similar function, as trail pheromones have been proposed to play a role in colonising new habitats in this species [241], although the responsible molecules have not been identified yet. Since we only analysed males, a study of both sexes in this and other species would help to clarify whether the composition of olfactory lobes exhibits sexual dimorphism and interspecific variation among velvet worms.

In arthropods, olfactory information is processed in the olfactory lobes and forwarded via projection neurons to mushroom body calyces and a neuropil called lateral horn [246–249]. A connection between olfactory lobes and mushroom bodies has also been reported in onychophorans [27, 31–33], albeit previous studies described an association with the calyces, which we did not detect. Instead, each olfactory lobe of *E. rowelli* is associated with four fascicles, only the thickest of which—the olfactory fascicle 4—is connected to the lobes of the mushroom body, whereas the olfactory fascicles 1–3 extend towards the median part of the brain (Fig. 13). This suggests that, as in insects, olfactory information passes to at least two higher-order processing centres, but the organisation of these connections bears only sparse resemblance to the situation in insects. As proposed for insects [250], the presence of multiple olfactory centres in the onychophoran brain might be related to the need for processing memory-related and memory-unrelated olfaction. This hypothesis remains to be substantiated by physiological and behavioural experiments.

### Potential homologues of onychophoran antennae across panarthropods

The segmental identity of the anteriormost appendages of onychophorans (“primary antennae” *sensu* Scholtz and Edgecombe [251]) and their potential homologues in other panarthropods have been subject to controversy (e.g. [13, 31, 34, 45, 47, 58, 75, 79, 252–261]). In the last two decades, considerable progress has been made on two issues related to this controversy:

1. It is likely that the onychophoran antennae are serially homologous with trunk limbs. This is suggested in onychophorans by the occurrence of a transitory nephridial anlage at the antennal basis, similar to the segmental nephridia in the leg-bearing body regions [124, 262], as well as corresponding expression patterns of appendage patterning genes in the antennae and other limbs [263].
2. Head development [34], neuronal tracing [15] and gene expression data [16, 116, 168, 263] revealed that the onychophoran antennae belong to the anteriormost (ocular or protocerebral) segment. This corroborates the hypothesis that the onychophoran antennae are not homologous with the first antennae of mandibulates (“secondary antennae” *sensu* Scholtz and Edgecombe [251, 255]) or the chelicerae of chelicerates, which are both innervated by the deutocerebrum and instead correspond to the onychophoran jaws [15, 168].

**Fig. 14:**
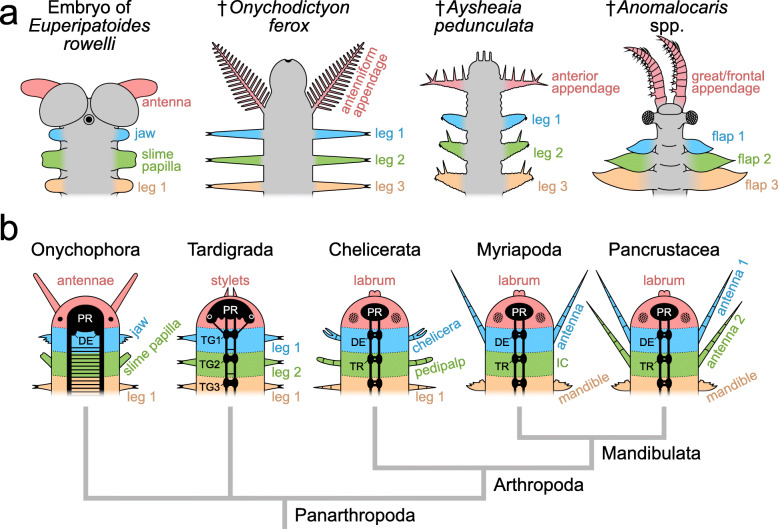
Alignment of cephalic segments and appendages across panarthropods. **a** Homology of anterior appendages in onychophorans and Cambrian lobopodians as proposed by Ou et al. [256] and Legg and Vannier [264]. Diagrams of †*O. ferox* †*A. pedunculata* and †*Anomalocaris* spp**.** after [256], [265], and [264, 266], respectively. Anterior end of stage IV embryo of *E. rowelli* redrawn from [59] to illustrate the position of developing limbs prior to their reorganisation and incorporation into the head. **b** Alignment of anterior segments with corresponding appendages in onychophorans, tardigrades and arthropods. Combined after [15, 59, 85, 251, 255, 259, 260]. Note that the condition illustrated for myriapods is also found in hexapodes within Pancrustacea. DE, deutocerebrum; IC, intercalary segment (without appendages); PR, protocerebrum; TG1, TG2, TG3, trunk ganglia 1 to 3; TR, tritocerebrum

The question of whether or not there are protocerebral homologues of the onychophoran antennae in living arthropods is difficult to address. The prevalent hypothesis considers the labrum of arthropods as the most likely candidate ([168, 259, 261]; Fig. 14b), as evidenced by its paired, appendicular origin in the protocerebral segment [267–273]. An alternative hypothesis homologises the onychophoran antennae with the frontal filaments of crustaceans [58, 257]. Although it seems plausible that frontal filaments are homologous across crustaceans [274], the appendicular nature of these structures is still questionable and casts doubt on their status as putative homologues to the onychophoran antennae [259, 261].

Given their corresponding position in the ocular/protocerebral segment, the onychophoran antennae might in fact be homologous with the anteriormost appendages of Cambrian stem-lineage onychophorans or (pan)arthropods, such as †*Aysheaia pedunculata* [265], †*Onychodictyon ferox* [256] and †*Antennacanthopodia gracilis* ([275]; Fig. 14a) as well as the onychophoran-like †*Illyodes inopinata* [276, 277] and †*Antennipatus montceauensis* [9] from the Carboniferous. The same holds true for their potential homology with the stylets of extant tardigrades, which might represent internalised limbs of the protocerebral segment [85, 256, 278, 279]; Fig. 14b).

The frontalmost limbs of various stem-group arthropods called great appendages or frontal appendages have also been homologised with the onychophoran antennae (e.g. [45, 256, 259–261]). However, their homology is uncertain even across arthropods, with divergent views regarding their correspondence to either primary [280, 281] or secondary antennae [261, 282–284]. An alternative recent hypothesis [255, 259, 260, 285] suggests that the frontal appendages of early-branching, stem-lineage arthropods, including representatives of †*Kerygmachela*, †*Opabinia* and †Radiodonta, might have been protocerebral structures homologous with the onychophoran antennae (Fig. 14a) and the labrum of arthropods, whereas the short great appendages of †Deuteropoda might have been associated with the deutocerebrum and are thus homologous with the chelicera/first antenna of arthropods.

A particular case is represented by the frontal appendage of the radiodont †*Lyrarapax unguispinus*. It has been interpreted as a pre-protocerebral structure associated with a pre-protocerebral ganglion rather than with the brain itself [45, 75]. The authors also reported a similar arrangement in the onychophoran *E. rowelli*, concluding that the antennal nerve cords (“frontal appendage nerve” *sensu* Cong et al. [45]) are associated with pre-protocerebral ganglia. The complete reconstructions of brain neuropils presented herein revealed no evidence for such ganglia in *E. rowelli*, as also demonstrated by previous immunolabelling and dye fill data [15]. Irrespective of whether the frontal appendages of †*L. unguispinus* are pre-protocerebral [45] or protocerebral, as in other radiodonts [260, 285], we conclude that frontal ganglia cannot be used to homologise the antennae of †*L. unguispinus* and *E. rowelli*.

### Open questions regarding the organisation of visual neuropils

The optic tracts (*sensu* [15, 32, 33]) or optic neuropils (this study) have been subject of ongoing debates regarding homology and evolution of visual organs and their associated brain centres across panarthropods [33, 42, 44, 69]. The presence of second-order visual neuropils flanking the central body as well as a connection of the optic neuropil to the mushroom body has led to the assumption that the nervous system of onychophorans might be more similar to that of chelicerates than other arthropods [42]. Our datasets did not reveal a connection with the mushroom bodies, suggesting this is an autapomorphy of chelicerates rather than a synapomorphy of chelicerates and onychophorans. While our results confirm connectivity between the optic neuropil and the central body [30–32, 54], we found no evidence of a “second optic neuropil” in *E. rowelli* [42], in which optic fibres would terminate. The optic neuropil with all its branches instead represents a unitary structure, which connects to the posterior division of the central body (Fig. 13), suggesting that visual signals are directly relayed by first-order interneurons into this higher-order processing centre. This again contrasts with the situation in arthropods, in which both median ocelli and compound eyes are associated with a second-order visual neuropil [169, 172, 286, 287]. The absence of second-order neuropils may indicate that these were either lost in Onychophora, assuming that they were already present in the last common ancestor of Panarthropoda, or that they first arose in the arthropod lineage. Reconstructing the visual system [288] and, in particular, the optic connectivity in tardigrades could help to clarify this issue.

The proposed connection of the optic neuropil to the mushroom body in *E. rowelli* [33] still requires corroboration, as we were unable to reconstruct all branches of the visual pathway in this species. A similar connection has been reported from the secondary eyes (i.e. derivatives of compound eyes) of spiders [33,83, 143, 149]. This would suggest homology of the onychophoran eyes with the compound eyes [33] rather than the median ocelli of arthropods [44]. The use of more specific methods, such as neuronal tracing [39, 40], in conjunction with 3D reconstruction might help to demonstrate the connectivity of the visual system in onychophorans and clarify the homology of visual organs and their neuropils across panarthropods.

## Conclusions

The present multiscale approach comprising 3D reconstruction revealed unprecedented details of the onychophoran brain, including the shape, position, spatial relationship and relative volume and size of individual tracts, lobes and neuropils. We have shown here that each mushroom body of *E. rowelli* is comprised of four rather than three lobes and that the accessory stalks [32] are not part of the mushroom bodies, but rather independent commissures associated with the antennal and subantennal neuropils. Despite detailed analyses using multiple methods, we were unable to endorse the existence of a frontal ganglion in the brain of *E. rowelli* [45], further questioning its proposed presence in onychophorans [47].

Our data further revealed a higher complexity of the onychophoran central body than previously reported, as this midline neuropil not only comprises anterior and posterior divisions (occurring only dorsally) but is additionally stratified into an inner and an outer lamina exhibiting an inverted arrangement in the two divisions and surrounding a pair of cylindrical auxiliary neuropils. Using 3D reconstruction, we characterised in detail the olfactory lobes and the individual glomeruli and, in addition, identified novel microglomerular structures, the function of which remains unknown. Most importantly, we identified a largely neglected anterior neuropil called the frontal body, which is one of the most prominent structures of the onychophoran brain. The previously suggested homology of the frontal body (“bridge” *sensu* [32]) with the protocerebral bridge of pancrustaceans is not well substantiated and therefore doubtful.

In summary, the present study improves our understanding of the anatomy and evolution of the onychophoran brain. It also clarifies several controversies surrounding the existence and composition of specific cerebral structures and their connectivity, thus shedding light on their evolutionary origins and homology. Our improved, refined and expanded nomenclature of neuroanatomical terms (Table 1) may serve as a basis for future studies of onychophoran neuroanatomy.

## Methods

### Collection and maintenance of specimens

Specimens of *Euperipatoides rowelli* Reid, 1996 were obtained from decaying logs and leaf litter in the Tallaganda State Forest (New South Wales, Australia; 35° 30′ 31″ S, 149° 36′ 14″ E, 934 m) in October 2016. They were collected under the permit number SPPR0008 issued by the Forestry Commission of New South Wales and exported under the permit number WT2012-8163 provided by the Department of Sustainability, Environment, Water, Population and Communities. The animals were maintained in culture as described elsewhere [289]. Only male specimens were used for the present study.

### Synchrotron radiation-based X-ray micro-computed tomography

For synchrotron radiation-based X-ray micro-computed tomography (SRμCT), two specimens were prepared following the protocol of Jahn et al. [87]. The first specimen, used for reconstructing the brain and the nerve cords, was anaesthetised in chloroform vapour for 10 s, fixed overnight in glutaraldehyde (2.5 % in 0.1 M sodium cacodylate buffer), washed several times in cacodylate buffer and stained overnight in 2% osmium tetroxide (OsO_4_, 4 %, Carl Roth, Karlsruhe, Germany). For the second specimen, used for reconstructing the anterior neuropils, ruthenium red (RR) was used as a staining agent, as it has proven useful for visualising the nervous system of onychophorans using SRμCT [87]. The specimen was first fixed and stained for 2 h in a solution containing 4% paraformaldehyde (PFA) and 0.3% RR (Carl Roth) in phosphate-buffered saline (PBS) and then decapitated, after which the head was left in the same solution for 5 days. Both specimens were washed several times in the corresponding buffers. Thereafter, the RR-stained specimen was kept in buffer, whereas the OsO_4_-stained specimen was dehydrated through an ascending acetone series (30, 50, 70, 90, 95, 3 × 100% in H_2_O; 15 min each) and embedded in Araldite® (Huntsman Advanced Materials, Salt Lake City, UT, USA). For SRμCT analyses, the Araldite®-block containing the OsO_4_-stained specimen was glued onto a standardised sample holder (HZG, Geesthacht, Germany), whereas the RR-stained head was scanned in a small container, which was filled with PBS and then glued onto a sample holder. Specimens for SRμCT were scanned at the beamline P05 of the storage ring PETRA III (Deutsches Elektronen-Synchrotron – DESY, Hamburg, Germany) operated by the Helmholtz-Zentrum hereon [290, 291] as described by Jahn et al. [87] with the following modification: the RR-stained specimen was scanned using inline grating differential phase-contrast (=PBCC) with a photon energy of 20 keV. We have applied a transport of intensities (TIE) phase retrieval approach to the recorded projections prior to the tomographic reconstruction in a custom-implemented reconstruction pipeline [292] using Matlab (Math-Works) and the ASTRA toolbox [293–295]. For the processing, raw projections were binned two times, which resulted in an effective voxel size in the final tomographic reconstructed volume of 2.4 μm for the RR-stained specimen, and 4.92 μm for the OsO_4_ stained specimen.

### Histology and light microscopy

For histology, specimens were anaesthetised in chloroform vapour, fixed in alcoholic Bouin’s fluid (Dubosq-Brasil; see [296]) for 2 days and washed in 70% ethanol. After dehydration through an ascending ethanol series (70%, 80%, 90%, 95%, 2 × 100%; 2 h each), specimens were incubated for 18 h in methyl benzoate and 24 h in 1-butanol and then embedded in Paraplast Plus® (Sherwood Medical Company, St. Louis, MO, USA) through a series of 1-butanol:Paraplast Plus® mixtures (2:1, 1:2 and then pure Paraplast Plus®) for 24 h each at 60 °C. A complete series of 5-μm sections was generated using a Leitz 1516 microtome (Ernst Leitz, Wetzlar, Germany). The obtained sections were stained with Heidenhain’s Azan staining method ([297]; modified by Geidies [298]) and analysed under an Axio Imager M2 light microscope (Carl Zeiss Microscopy, Jena, Germany) equipped with an Axiocam 503 colour digital camera (Carl Zeiss Microscopy).

### Immunohistochemistry and confocal laser scanning microscopy

To obtain details of individual tracts and neuropils, neural markers against acetylated α-tubulin and synapsin were used in conjunction with confocal laser scanning microscopy (CLSM). Synapsin occurring in synaptic terminals was labelled using the monoclonal anti-synapsin serum (Developmental Studies Hybridoma Bank, Iowa City, IA, USA; DSHB Product 3C11 [antiSYNORF1]) raised in mouse against a fusion protein consisting of glutathione-S-transferase and the SYN1 protein of *Drosophila melanogaster* [299]. Acetylated α-tubulin was targeted using a monoclonal antibody (Sigma-Aldrich, St. Louis, MO, USA, cat. nr. T7451) raised in mouse against acetylated α-tubulin from the outer arm of the sea urchin *Strongylocentrotus purpuratus*. The antibodies against synapsin and acetylated α-tubulin have both been shown to recognise neuropil and neurite regions in the nervous systems of various invertebrates, including onychophorans, tardigrades and non-insect arthropods (e.g. [3, 15, 18, 69, 167, 169, 235, 300]). The staining protocol was based on the technique developed by Ott [301].

For whole-mount labelling of brains, specimens were anaesthetised in chloroform vapour and dissected in HEPES-buffered saline (HBS). Subsequently, the dissected brains were fixed overnight in zinc formaldehyde (ZnFA: 18.4 mM ZnCl_2_, 135 mM NaCl, 35 mM sucrose, 1% formaldehyde), briefly washed in HBS, and treated with a mixture of collagenase/dispase (RocheDiagnostics, Mannheim, Germany; 1 mg/mL each) and hyaluronidase (Sigma-Aldrich; 1 mg/mL) in Tris-buffered saline (TBS; 50 mM Tris, 150 mM NaCl, pH 7.4) for 40 min at 37 °C. After several rinses in TBS, the samples were incubated in a solution containing 80% methanol and 20% dimethyl sulfoxide (DMSO) for 1.5 h, followed by an incubation in 100% methanol. The samples were then rehydrated through a descending methanol series (90%, 70%, 50%, 30% in TBS; 10 min each) and blocked in 5% normal goat serum (NGS; Sigma-Aldrich) in TBS containing 1% DMSO (TBS-D) for 1 h. Thereafter, the brains were incubated with the primary synapsin and acetylated α-tubulin antisera (1:50 and 1:500, respectively; in TBS-D, 1% NGS) for 14 days at 4 °C. After several washes in TBS-D, the samples were incubated for 10 days with the secondary antibody (goat anti-mouse, Alexa Fluor® 488; Thermo Fischer Scientific, Waltham, MA, USA; 1:500 in TBS-D, 1% NGS). To control for non-selective staining due to the secondary antibody, we ran another protocol omitting the primary antibody, which resulted in no signal. After several washes in TBS-D and then TBS, the brains were counterstained with 4′,6-diamidino-2-phenylindole (DAPI; Carl Roth; 1 ng/mL in TBS) for 1 h, rinsed several times in TBS, dehydrated through an ascending ethanol series (70%, 90%, 95%, 100%, 100%, in TBS; 10 min each), and finally cleared and mounted between two coverslips in methyl salicylate using a carbon fibre reinforced polymer slide as spacer.

Additional anti-synapsin labelling was conducted on vibratome sections. For this, specimens were anaesthetised in chloroform vapour and fixed in ZnFA overnight. After several rinses in HBS, specimens were cut in halves and embedded in a solution containing 3.75 g albumin from chicken egg (Sigma-Aldrich) and 0.5 g gelatine from porcine skin (Type A; Sigma-Aldrich) in 12 mL distilled water. Albumin-gelatine blocks were fixed in 10% PFA overnight and cut in 100-μm-thick sections using a vibratome (MICROM 650 V, Microm International, part of Thermo Fisher Scientific, Walldorf, Germany). Obtained sections were subsequently labelled as for whole-mount samples with the following modifications: (i) dehydration and rehydration steps were omitted, and (ii) the sections were mounted between two coverslips in ProLong Gold^TM^ Antifade Mountant (Invitrogen, Carlsbad, CA, USA).

Both whole-mount preparations and vibratome sections were analysed using a confocal laser scanning microscope (Zeiss LSM 880; Carl Zeiss Microscopy) equipped with an Airyscan module. Images were acquired using the ZEN 2 (black edition) imaging software (Carl Zeiss Microscopy). The images were taken using 488 nm (for the secondary antibody) and 405 nm lasers (for DAPI) with the emission wavelengths of 516 nm and 450 nm, respectively.

### Data and image processing

Three-dimensional reconstructions were conducted using the following five datasets. Stacks of SRμCT images were used for reconstructing (i) the overall structure of the central nervous system as well as (ii) its anterior part subdivided in perikaryal and neuropil regions*.* After manual segmentation of structures of interest using Amira 6.0.1 (FEI Visualization Sciences Group, Burlington, MA, USA), label fields were separated using Fiji v.1.52 [302, 303]. (iii) Series of histological micrographs were used for reconstructing the anterior nervous system. For this, digitised images were aligned and target structures segmented and reconstructed using Amira 6.0.1. Finally, CLSM stacks of entire brains labelled with DAPI and antibodies against (iv) synapsin and (v) acetylated α-tubulin were used for reconstructing individual tracts, cell layers and neuropils. Raw Airyscan datasets were processed using the ZEN 2 software (black edition) and handled further with Amira 6.0.1 as for the other datasets. Three-dimensional volume and surface renderings of SRμCT, histological and CLSM data were carried out with VGStudio MAX 3.0 (Volume Graphics, Heidelberg, Germany).

Relative volume estimates of brain neuropils were conducted taking into account the number of voxels in each segmented structure relative to the number of voxels in the entire brain. Potential shrinkage artefacts (e.g. [304, 305]) were not considered, as we expect that the structures (i.e. nervous tissue) shrink relative to each other, resulting in similar or even the equal volume ratios. Prior to the relative volume estimates, the brain was segregated from the remaining parts of the central nervous system, such as the antennal nerve cords, circumpharyngeal nerve cords and ventral nerve cord, using the Regions of Interest function (ROI) in VGStudio MAX 3.0. Raw datasets were processed (Airyscan mode: Superresolution) using the ZEN 2 software (black edition). Selected substacks of entire brains and vibratome sections were adjusted for optimal brightness and contrast in Fiji v.1.52. Final panels and diagrams were compiled with Adobe Illustrator CS5.1 (Adobe Systems, San Jose, CA, USA).

## Supplementary Information

**Additional file 1: Figure S1.** General organisation and position of the brain in *E. rowelli*. Three-dimensional reconstruction based on SRμCT dataset in anterolateral (**a**) dorsal (**b**) and posterior views (**c**). Dorsal is up in (**a** and **c**), anterior is up in (**b**). A cell body rind (blue) partly covers the neuropils (light-brown). Arrows point to somata-free regions. ANC, antennal nerve cord; CNC, circumpharyngeal nerve cord; DE, deutocerebrum; EY, eye; HO, hypocerebral organ; PR, protocerebrum; SPC, subpharyngeal commissure; VC, ventral commissure; VNC, ventral nerve cord

**Additional file 2: Figure S2.** Organisation of fascicles of mushroom body in *E. rowelli*. Horizontal optical sections from CLSM stack of brain. Anterior is up in all images. **a** DNA staining, **b** anti-acetylated α-tubulin immunoreactivity, and **c** merged. Note that fascicles (dotted line) are associated with dense accumulation of small somata (arrowheads) of globuli cells and contains four major, branched fascicles (arrows). ANP, antennal neuropil; CBR, cell body rind; OLF, olfactory lobe; PED, pedunculus. Scale bar: 100 μm

**Additional file 3: Figure S3.** Organisation and position of brain commissures 1 and 2 in *E. rowelli*. Greyscale light micrographs of selected series of Azan-stained histological sections through the head (from ventral to dorsal). Anterior is up in all images. **a**–**f** Pathway of brain commissures 1 (arrowheads) and 2 (arrows). Asterisks indicate anterior parts of four mushroom body lobes. LO, lobes of the mushroom body; OLF, olfactory lobe; PVN, posterior ventral neuropil. Scale bars: 200 μm

**Additional file 4: Figure S4.** Details of olfactory glomeruli and microglomeruli in *E. rowelli*. Optical sections of confocal micrographs from anti-synapsin immunolabelled vibratome cross sections. **a** Overview of single glomerulus from olfactory lobe. **b** Detail from (**a**) illustrating synapsin-immunoreactive sites. **c** Overview of single microglomerulus. **d** Higher magnification of (**c**) exhibiting synapsin-immunoreactive sites. Note numerous synaptic terminals in both, glomerulus and microglomerulus. (**a** and **c**) and (**b** and **d**) are to scale. Scale bars: 5 μm (**a** and **c**) and 1 μm (**b** and **d**)

**Additional file 5: Figure S5.** Organisation and characterisation of brain neuropils in *E. rowelli*. Three-dimensional reconstruction based on anti-acetylated α-tubulin immunolabelling. Brain in frontal (**a** and **d**), anterodorsal (**b**), and posteroventral views (**c**) illustrating largely fused neuropils and lobes (bluish grey). **a** and **b** For clarity the mushroom bodies, olfactory lobes, frontal neuropil, visual pathways, and central body are collectively shown in semi-transparent light grey. ANC, antennal nerve cord; ANP, antennal neuropil; CNC, circumpharyngeal nerve cord; CN, central neuropil; DN, deutocerebral neuropil; FB, frontal body; FGC, fascicles of globuli cells; JAN, jaw nerve; LIN 2, lip nerve 2; ML, median lobe; OFG, olfactory glomeruli; ONP, optic neuropil; PDN, posterior dorsal neuropil; PHN 1 pharyngeal nerves 1; PVN, posterior ventral neuropil; SAN, subantennal neuropil; SPC, subpharyngeal commissure; SPN, anterior and posterior slime papilla nerves; VC, ventral commissures; VH, ventral horn

**Additional file 6: Data S1.** Image stack showing SRμCT scan of *E. rowelli* head used for segmentation

**Additional file 7: Data S2.** Image stack showing aligned greyscale light micrographs of Azan-stained sections of *E. rowelli* head used for segmentation

**Additional file 8: Data S3.** CLSM stack of *E. rowelli* brain labelled against acetylated α-tubulin, scanned from ventral side and used for segmentation

**Additional file 9: Data S4.** CLSM stack of *E. rowelli* brain labelled against acetylated α-tubulin, scanned from dorsal side and used for segmentation

**Additional file 10: Data S5.** CLSM stack of *E. rowelli* brain labelled against synapsin, scanned from ventral side and used for segmentation

**Additional file 11: Data S6.** CLSM stack of *E. rowelli* brain labelled against synapsin, scanned from dorsal side and used for segmentation

## Data Availability

All data generated or analysed during this study are included in this published article, its supplementary information files and publicly available within the figshare repository: 10.6084/m9.figshare.14899185.

## Abbreviations

a: Anterior
ANC: Antennal nerve cord
ANP: Antennal neuropil
BR: Brain
BRC 1, BRC 2, BRC 3: Brain commissure 1 to 3
CB: Central body
CBA: Anterior division of central body
CB-AN: Auxiliary neuropil of central body
CB-IL: Inner lamina of central body
CB-OL: Outer lamina of central body
CBP: Posterior division of central body
CBR: Cell body rind
CN: Central neuropil
CNC: Circumpharyngeal nerve cord
d: Dorsal
DC: Dorsal commissures
DE: Deutocerebrum
DN: Deutocerebral neuropil
δ: Dorsal or δ-lobe of mushroom body
E-ONP: Extracerebral part of optic neuropil
EY: Eye
FB: Frontal body
FGC: Fascicles of globuli cells
HO: Hypocerebral organ
IC: Intercalary segment (without appendages)
I-ONP: Intracerebral part of optic neuropil
JAN: Jaw nerve
λ: Lateral or λ-lobe of mushroom body
LE: Leg
LEN: Leg nerve
LIN 1, LIN 2, LIN 3: Lip nerve 1 to 3
LO: Lobes of mushroom body
MG: Macroglomerulus
MIG: Microglomeruli
ML: Median lobe
μ: Median lobe or μ-lobe of mushroom body
OFG: Olfactory glomeruli
OLF 1, OLF 2, OLF 3, OLF 4: Olfactory fascicle 1 to 4
OLF: Olfactory lobe
OLG: Olfactory glomeruli
OLG: Olfactory glomeruli
OLL: Olfactory lobe
ONP: Optic neuropil
p: Posterior
PDN: Posterior dorsal neuropil
PED: Pedunculus
PHN 1, PHN 2: Pharyngeal nerves 1 and 2
PNT: Ponticulus
PR: Protocerebrum
PVN: Posterior ventral neuropil
SAN: Subantennal neuropil
SGN: Salivary gland nerve
SP: Slime papilla
SPC: Subpharyngeal commissure
SPN: Anterior and posterior slime papilla nerves
SRC: Suprarectal commissure
TG1, TG2, TG3: Trunk ganglia 1 to 3
TR: Tritocerebrum
v: Ventral
ν: Ventral or ν-lobe of mushroom body
VC: Ventral commissure
VH: Ventral horn
VNC: Ventral nerve cord
