# Supplementary figures and images for: The velvet worm brain unveils homologies and evolutionary novelties across panarthropods
Source: BMC Biol. 2022 Jan 25;20:26. doi: 10.1186/s12915-021-01196-w (PMC9136957; doi:10.1186/s12915-021-01196-w)

a

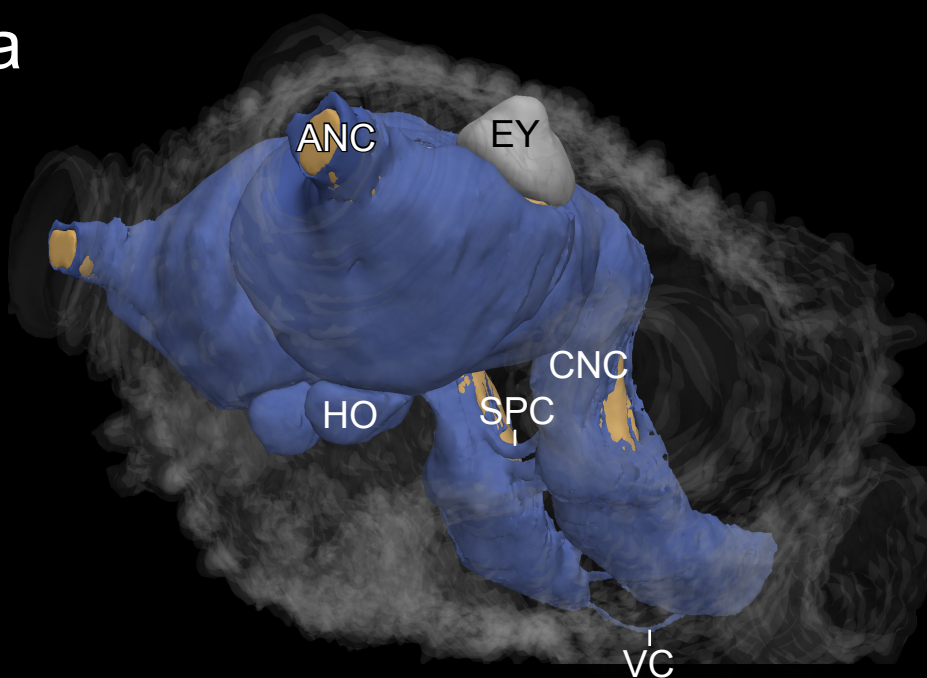

b

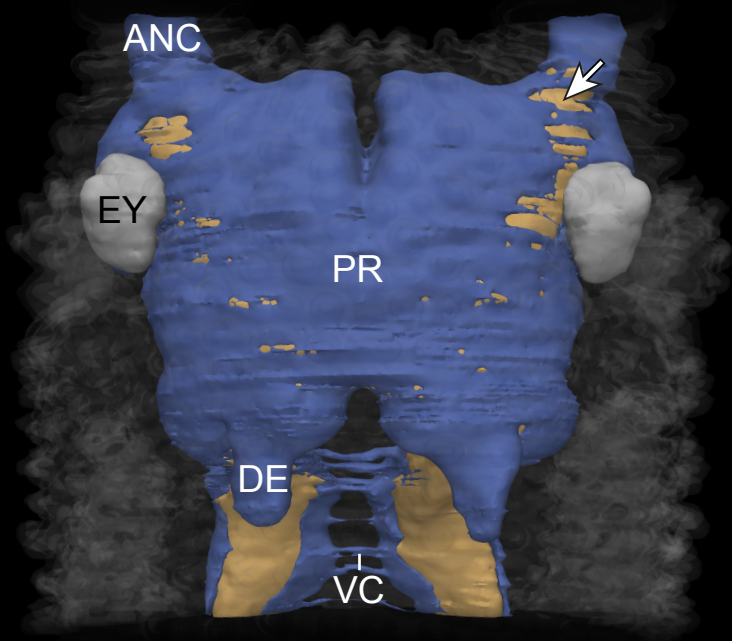

c

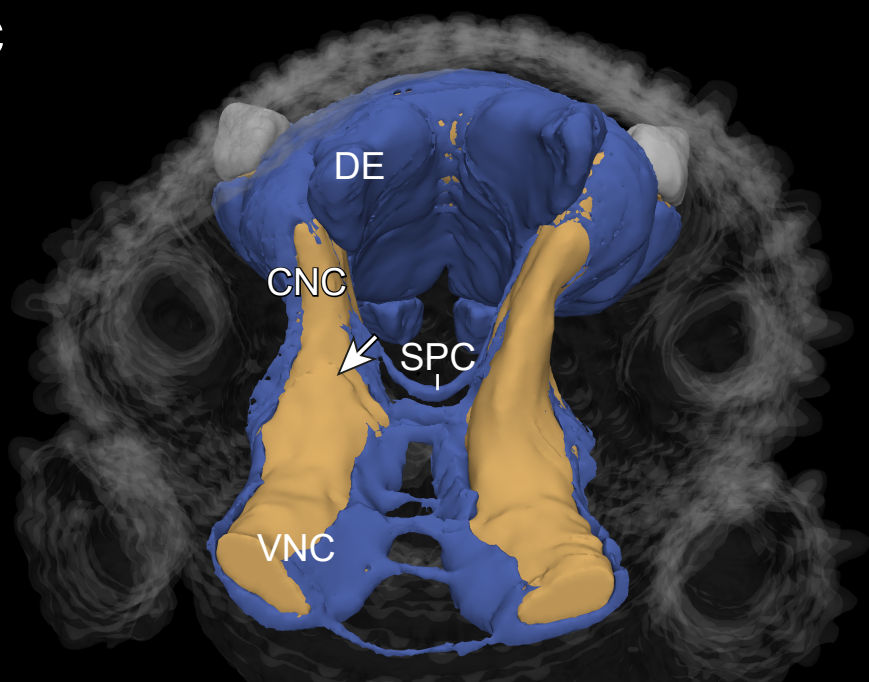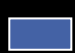

cell body rind

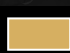

neuropil

Supplement: Supplementary file 1 — Additional file 1: Figure S1. General organisation and position of the brain in E. rowelli. Three-dimensional reconstruction based on SRμCT dataset in anterolateral (a) dorsal (b) and posterior views (c). Dorsal is up in (a and c), anterior is up in (b). A cell body rind (blue) partly covers the neuropils (light-brown). Arrows point to somata-free regions. ANC, antennal nerve cord; CNC, circumpharyngeal nerve cord; DE, deutocerebrum; EY, eye; HO, hypocerebral organ; PR, protocerebrum; SPC, subpharyngeal commissure; VC, ventral commissure; VNC, ventral nerve cord [file 12915_2021_1196_MOESM1_ESM.pdf]

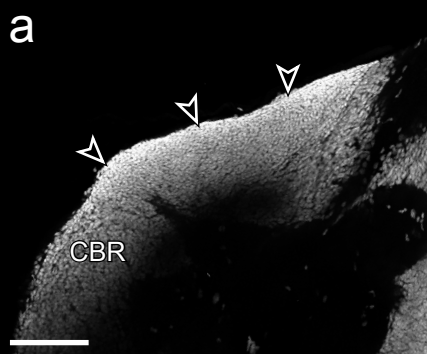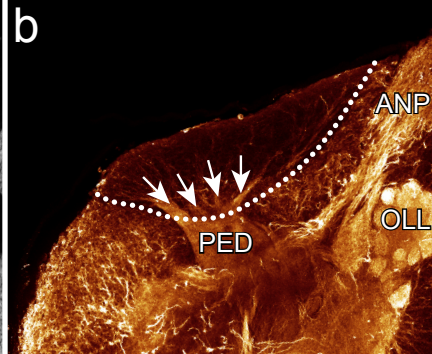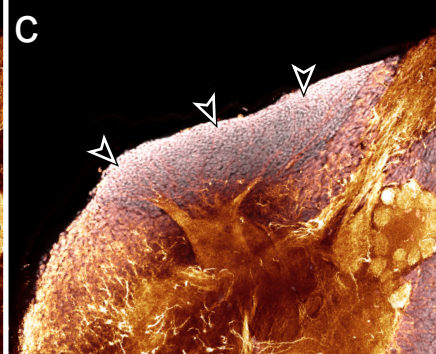

Supplement: Supplementary file 2 — Additional file 2: Figure S2. Organisation of fascicles of mushroom body in E. rowelli. Horizontal optical sections from CLSM stack of brain. Anterior is up in all images. a DNA staining, b anti-acetylated α-tubulin immunoreactivity, and c merged. Note that fascicles (dotted line) are associated with dense accumulation of small somata (arrowheads) of globuli cells and contains four major, branched fascicles (arrows). ANP, antennal neuropil; CBR, cell body rind; OLF, olfactory lobe; PED, pedunculus. Scale bar: 100 μm [file 12915_2021_1196_MOESM2_ESM.pdf]

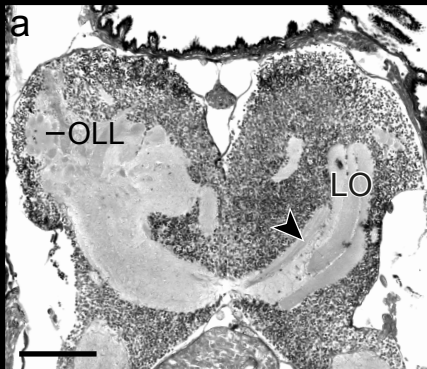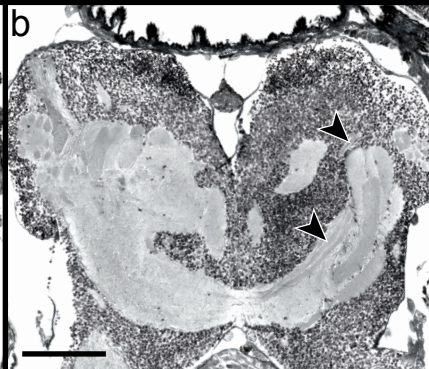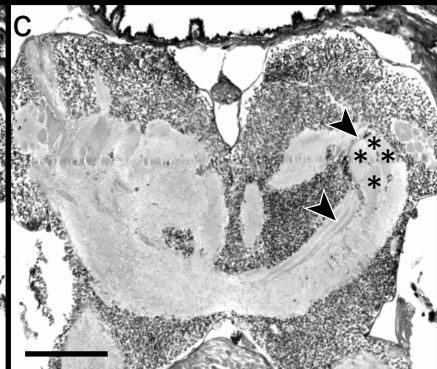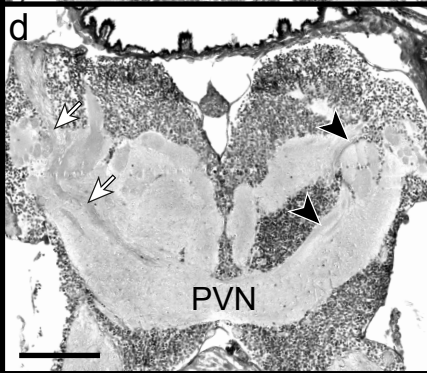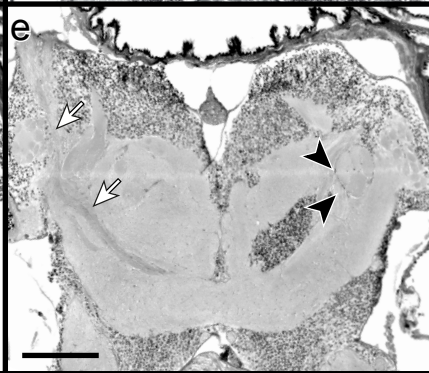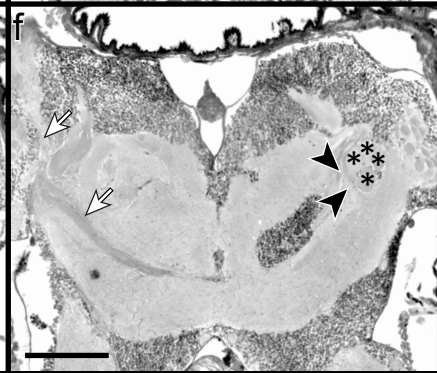

Supplement: Supplementary file 3 — Additional file 3: Figure S3. Organisation and position of brain commissures 1 and 2 in E. rowelli. Greyscale light micrographs of selected series of Azan-stained histological sections through the head (from ventral to dorsal). Anterior is up in all images. a–f Pathway of brain commissures 1 (arrowheads) and 2 (arrows). Asterisks indicate anterior parts of four mushroom body lobes. LO, lobes of the mushroom body; OLF, olfactory lobe; PVN, posterior ventral neuropil. Scale bars: 200 μm [file 12915_2021_1196_MOESM3_ESM.pdf]

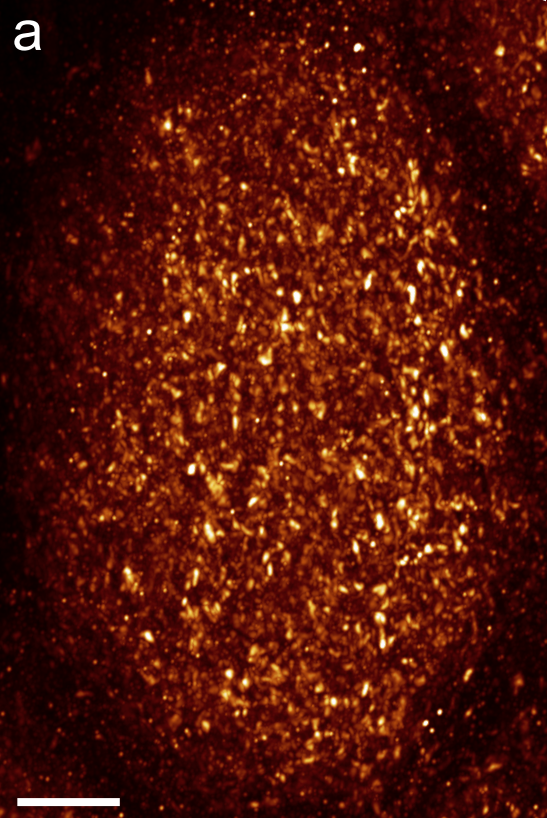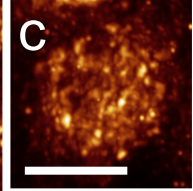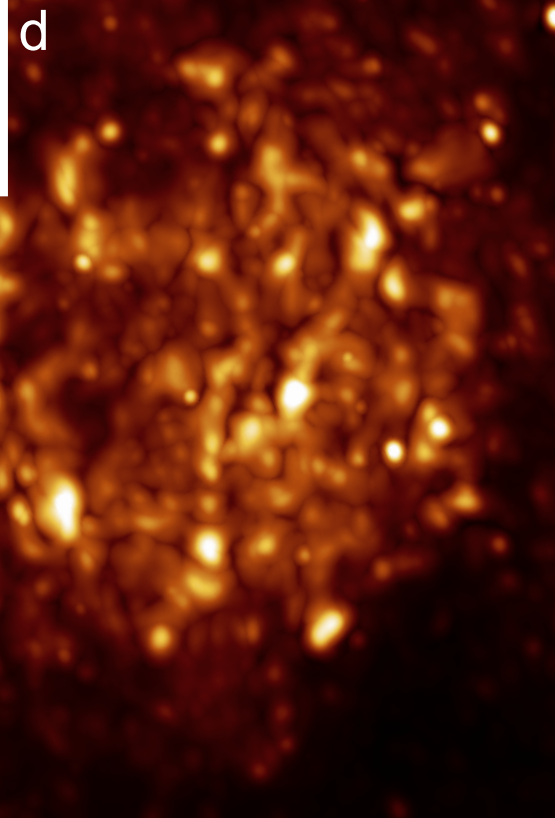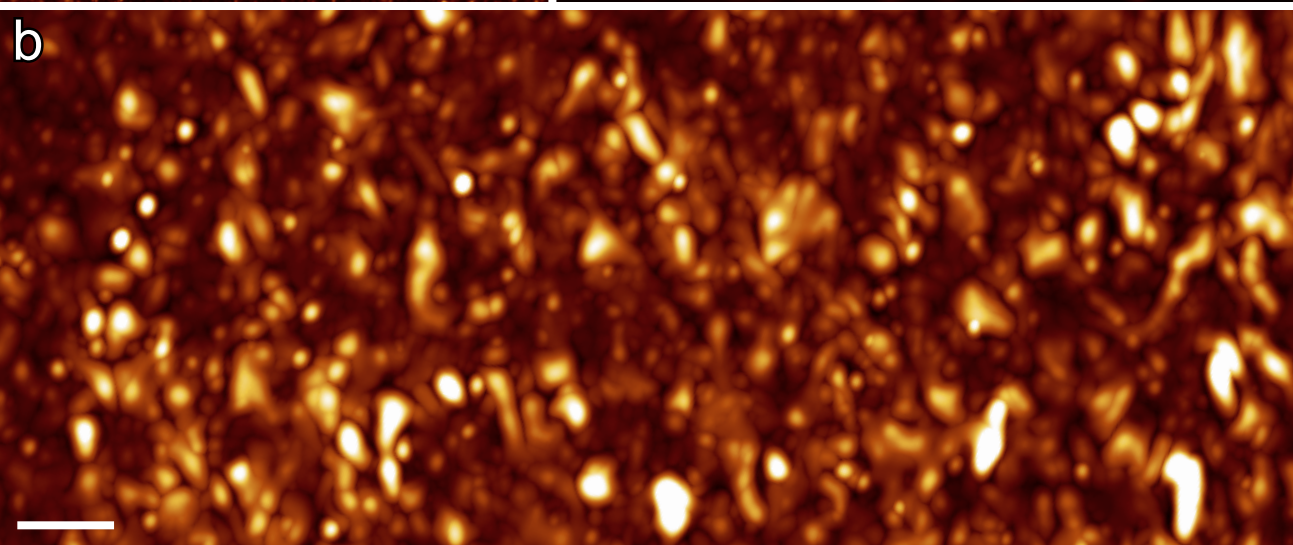

Supplement: Supplementary file 4 — Additional file 4: Figure S4. Details of olfactory glomeruli and microglomeruli in E. rowelli. Optical sections of confocal micrographs from anti-synapsin immunolabelled vibratome cross sections. a Overview of single glomerulus from olfactory lobe. b Detail from (a) illustrating synapsin-immunoreactive sites. c Overview of single microglomerulus. d Higher magnification of (c) exhibiting synapsin-immunoreactive sites. Note numerous synaptic terminals in both, glomerulus and microglomerulus. (a and c) and (b and d) are to scale. Scale bars: 5 μm (a and c) and 1 μm (b and d) [file 12915_2021_1196_MOESM4_ESM.pdf]

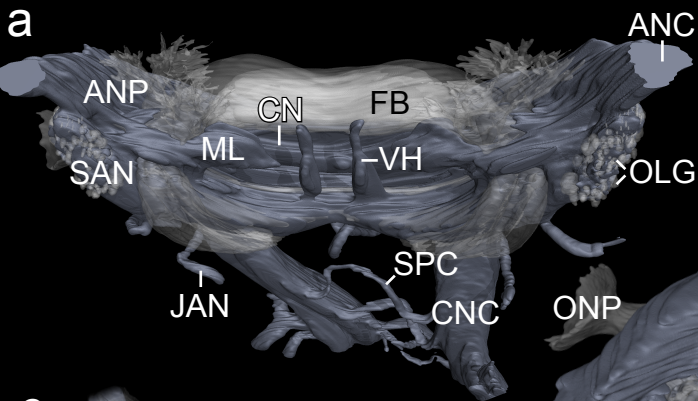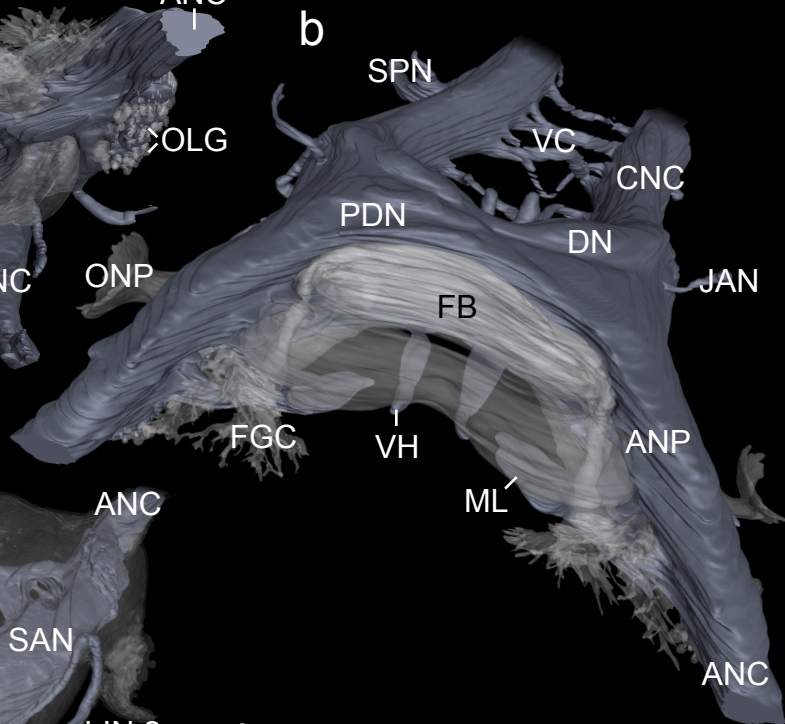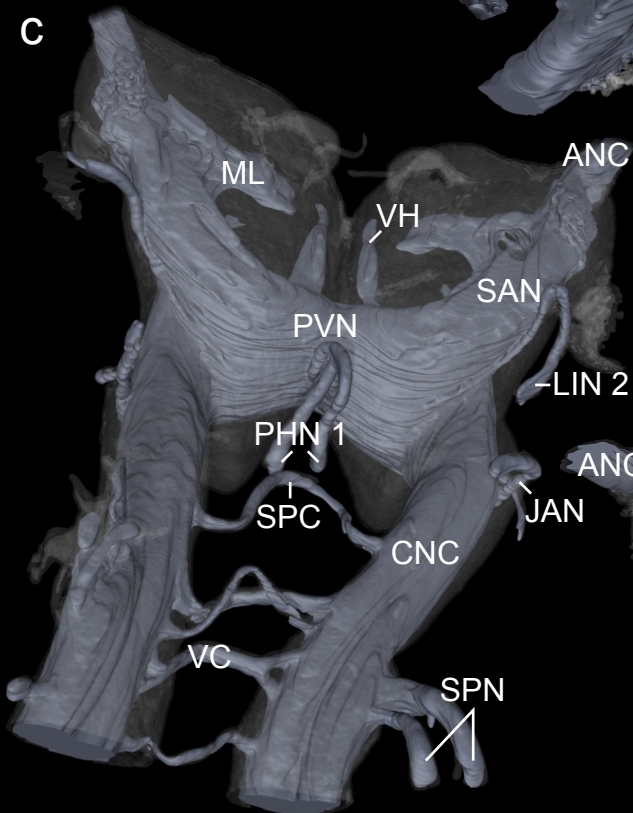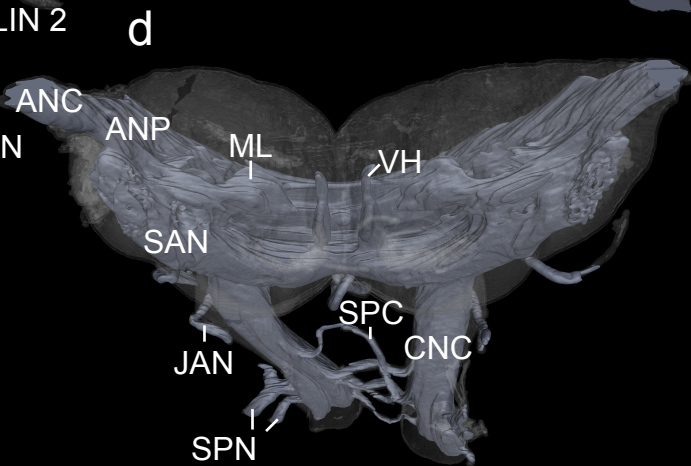

Supplement: Supplementary file 5 — Additional file 5: Figure S5. Organisation and characterisation of brain neuropils in E. rowelli. Three-dimensional reconstruction based on anti-acetylated α-tubulin immunolabelling. Brain in frontal (a and d), anterodorsal (b), and posteroventral views (c) illustrating largely fused neuropils and lobes (bluish grey). a and b For clarity the mushroom bodies, olfactory lobes, frontal neuropil, visual pathways, and central body are collectively shown in semi-transparent light grey. ANC, antennal nerve cord; ANP, antennal neuropil; CNC, circumpharyngeal nerve cord; CN, central neuropil; DN, deutocerebral neuropil; FB, frontal body; FGC, fascicles of globuli cells; JAN, jaw nerve; LIN 2, lip nerve 2; ML, median lobe; OFG, olfactory glomeruli; ONP, optic neuropil; PDN, posterior dorsal neuropil; PHN 1 pharyngeal nerves 1; PVN, posterior ventral neuropil; SAN, subantennal neuropil; SPC, subpharyngeal commissure; SPN, anterior and posterior slime papilla nerves; VC, ventral commissures; VH, ventral horn [file 12915_2021_1196_MOESM5_ESM.pdf]
